# Exploring barriers and facilitators to physical activity among children in Saudi Arabian schools: A qualitative study

**DOI:** 10.1371/journal.pone.0329600

**Published:** 2025-09-15

**Authors:** Abdullah Alalawi, Lindsay Blank, Elizabeth Goyder

**Affiliations:** 1 Al Qunfudah Health Sciences College, Umm Al-Qura University, Makkah, Saudi Arabia; 2 School of Medicine and Population Health, University of Sheffield, Sheffield, United Kingdom; 3 School of Medicine and Population Health, University of Sheffield, Sheffield, United Kingdom; University of Luzon, PHILIPPINES

## Abstract

**Background:**

The growing global burden of noncommunicable diseases, exacerbated by insufficient physical activity (PA), contributes to a range of adverse physical, mental, and social health outcomes. This is a significant concern in the Kingdom of Saudi Arabia (KSA), where cultural and environmental factors further limit opportunities for PA, particularly among schoolchildren in urban areas.

**Aim:**

This study explores the challenges and opportunities related to school-based PA to inform future interventions targeting schoolboys aged 13–15 years in KSA.

**Methods:**

Qualitative data were collected through semi-structured interviews with 16 school staff members, seven parents and workshops involving 37 male students from two contrasting schools (one private and one public), in Jeddah City, KSA. All participants were recruited via purposive sampling. Follow-up interviews were conducted with a total of nine school staff members at both schools. Data were analysed using thematic analysis to identify key patterns and insights.

**Results:**

This study found that PA among students was influenced by multiple factors. Individual-level motivators included enjoyment, competition, and perceived health benefits, while barriers included academic pressures and sedentary preferences. Interpersonal influences involved varying levels of support from parents, peers, and teachers. School-related factors such as the physical environment, facilities, and institutional policies also shaped PA availability. At the community-level, participants highlighted the potential for interventions that adapt international programmes to the local context.

**Conclusion:**

This study underscores the need for a multi-dimensional approach to promoting PA among adolescents in Jeddah. Stakeholders should consider piloting and adapting proven international interventions, addressing disparities in school resources, increasing the time allocated for PA within the curriculum, and raising parental awareness about the importance of PA. These steps are essential to creating a supportive environment that fosters improved public health and youth well-being in KSA.

## Introduction

Noncommunicable diseases (NCDs) are a growing global health concern, disproportionately affecting populations in the Arabic region, including KSA, where the prevalence of diabetes (10.9%) and obesity rates are among the highest globally [1,2]. Physical inactivity is a major contributor to the surge in NCDs, including heart disease, diabetes, and hypertension, necessitating urgent preventive interventions [3]. Regular PA is universally acknowledged as a key strategy to mitigate these health risks, with documented benefits including improved cardiovascular health, enhanced muscular strength, better mental health outcomes, and reduced risk of chronic conditions [4,5].

Establishing regular PA during childhood and adolescence is particularly critical, not only for its immediate physical and mental health benefits but also because habits formed during this period often continue into adulthood [6]. Lifelong engagement in PA significantly reduces the risk of chronic diseases and supports sustained well-being. Adolescence, therefore, represents a critical period for embedding healthy lifestyle patterns that extend into later life.

Despite these benefits, PA levels among children and adolescents in KSA remain significantly below global recommendations. A systematic review [1] of studies published between 2007 and 2017 found that the prevalence of PA among Saudi adolescents ranged from 4% to 44.5%, indicating a substantial portion of this population is insufficiently active. Cultural, environmental, and systemic barriers including curriculum overload, insufficient facilities, and limited awareness have exacerbated sedentary lifestyles and contributed to rising obesity rates among Saudi youth [7,8].

Schools represent a critical setting for promoting PA, as they provide a structured environment where children spend a significant portion of their day. Evidence suggests that school-based PA interventions can effectively increase PA levels, improve health outcomes, and instill healthy habits [9,10]. Globally, initiatives like The Daily Mile (TDM) and classroom-based active breaks have demonstrated success in integrating PA into school routines, but these programmes often require adaptation to local contexts [11,12]. In KSA, the implementation of PA initiatives remains fragmented and insufficiently evaluated, highlighting the need for culturally relevant, sustainable interventions [13].

Previous reviews [14,15] evaluated existing literature on the effectiveness of school-based interventions aimed at increasing PA levels among school children aged 6–18 years globally and in the Middle East and Arabic-speaking countries specifically. Based on the findings from both reviews, it is clear that there are numerous international studies on school-based PA interventions for increasing the rates of PA among students, but fewer studies have focused on Arabic countries and, in particular, KSA.

This study addresses these gaps by exploring the barriers and enablers of school-based PA among male adolescents aged 13–15 years in Jeddah, KSA. Using the Social-Ecological Model (SEM) as a framework [16], the research examines factors influencing PA at the individual, interpersonal, organisational, and community levels. By engaging with students, parents, and school staff, this study aims to provide actionable recommendations for developing effective and context-specific PA interventions that not only increase activity in the short term but also support the establishment of active lifestyles into adulthood.

## Methods

### Study design and setting

This study employed a case study design to explore the barriers and enablers of school-based PA, grounded in the interpretivist paradigm and following an inductive approach [17]. The research was conducted in two schools in Jeddah, KSA: one private school located in an affluent district, and one public school situated in a socioeconomically disadvantaged area.

The affluent area is characterised by well-maintained infrastructure, gated residential compounds, and private recreational facilities, although access may still be restricted by safety concerns or cultural norms. In contrast, the deprived area has limited public amenities, smaller residential streets, and few accessible green spaces or sports grounds. While both areas contain some designated spaces for PA, their accessibility, quality, and safety vary considerably.

The two schools were selected through purposive sampling from a pool of six schools within the same administrative region. Selection was based on the presence of basic PA facilities (e.g., a schoolyard or sports hall), willingness to participate, and alignment with the socio-economic contrast desired for the study. Schools that completely lacked PA infrastructure were excluded to ensure the study could focus on existing but underutilized opportunities for PA, rather than on total absence. However, we acknowledge that the lack of facilities is itself an important barrier and discuss this point in the study’s limitations and implications.

Jeddah was selected as the study site due to its large, diverse population and distinctive cultural and urban context. Previous studies have rarely examined both the barriers and enablers of school-based PA among adolescent boys in KSA [14]. This study adhered to the Consolidated Criteria for Reporting Qualitative Research (COREQ) to ensure transparency and rigor in reporting (S1 Table) [18].

### Participants and recruitment

Participants included three participant groups: school staff (teachers and administrators), students aged 13–15 years, and parents. Purposive sampling was used to recruit participants [19], ensuring their alignment with the study’s objectives. Inclusion criteria for students included being enrolled in the selected schools and obtaining parental consent. Teachers were required to be Saudi nationals, while parents were recruited during scheduled school meetings. All participants were recruited in person at the schools, with assistance from a designated teacher assistant.

#### Recruitment of schools.

After obtaining ethical approval from the University of Sheffield’s Research Ethics Committee and the Saudi Ministry of Education, the author (AA), with assistance from the Ministry, purposefully selected two contrasting schools: a public school in a deprived area and a private school in an affluent area. Selection criteria included the availability of school grounds and sports facilities.

Purposive sampling was used to recruit both schools and participants, in line with the study’s aims and objectives, to ensure the selection of relevant participants and appropriate settings for interviews and workshops [19].

#### Recruitment of school staff.

After obtaining approval from the Ministry of Education, the author (AA) met with school principals to seek their consent for including staff and students in the study. During these meetings, the study’s purpose and significance were discussed, along with potential challenges, such as the possibility of teachers being reluctant to participate. A pre-notification letter was displayed on school noticeboards to inform staff about the project. The author was granted approval to deliver oral presentations to staff at each school, highlighting confidentiality and anonymity. Information sheets and consent forms were provided to those interested in participating.

Interviews were scheduled based on staff availability, and only Saudi national male teachers employed at the selected schools were eligible. This criterion ensured a deeper exploration of Saudi teachers’ perspectives on the challenges and opportunities for school-based PA among male students, in accordance with Saudi Arabia’s education system, where male teachers are assigned to boys’ schools.

#### Recruitment of students.

After obtaining approval from school principals, the author visited classrooms with support from teacher assistance to explain the study to students. Information sheets and consent forms were distributed, and students were verbally invited to participate. Those interested had to sign the consent form, while parental consent was also required and facilitated through forms sent home with students.

A 20% oversampling strategy was used to account for potential parental refusals. Eligibility criteria included being enrolled in the selected schools, aged 13–15 years, and having both student and parental consent.

#### Recruitment of parents.

During the initial meeting with school principals, the researcher emphasised the importance of fathers’ perspectives in understanding the challenges and opportunities for school-based PA. Due to cultural norms in KSA, recruiting mothers was not feasible. One principal suggested using an upcoming school-parent communication meeting as an opportunity to engage fathers.

The author attended the meeting, explained the study’s significance, addressed confidentiality concerns, and distributed information sheets and consent forms to interested participants.

### Sampling strategy

This study employed a purposive sampling approach to ensure the selected participants had relevant experience and insights aligned with the study’s objectives. The sample included intermediate school staff (teachers, administrators, and support staff with at least two years of experience), male students (aged 13–15 years), and parents of students in this age group.

Participants were selected based on their direct involvement in the educational process, ensuring a range of diverse perspectives. To achieve data saturation, qualitative interviews were conducted until no new themes emerged, following recommendations from [20,21]. This methodological approach enhanced the rigour and validity of the study.

### Data collection

Data were collected through semi-structured interviews with school staff, parents, and workshops with male students. All interviews and workshops were conducted in Arabic and took place in person within school settings in Jeddah, KSA, between April 25 and July 31, 2022.

The interviews followed a semi-structured format, allowing participants to elaborate on their experiences regarding school-based PA. The workshops with students included discussion-based activities and creative exercises, such as drawing, to foster engagement and self-expression. The lead researcher (AA), a native Arabic speaker with expertise in PA research, conducted all interviews and workshops, ensuring a culturally sensitive and contextually relevant approach.

All interviews and workshops were audio-recorded with participants’ consent, transcribed verbatim, and subsequently translated into English for analysis. Data collection continued until data saturation was reached [20,21].

#### 1. Interviews.

Interviews were conducted with school staff and parents in private settings within the schools. A topic guide informed by the study’s objectives and a systematic review of existing literature was used [14] (see S1 Table). Questions explored participants’ views on barriers and enablers of PA, current practices, and recommendations for interventions.

Interviews lasted between 35 and 45 minutes, were audio-recorded with participants’ consent, and accompanied by note-taking.

#### 2. Workshops.

Workshops were conducted in two sessions at each school. The first session involved group discussions about students’ experiences with and perceptions of PA. The second session featured a creative drawing exercise, in which students illustrated perceived barriers and facilitators to PA.

Discussions were facilitated by the lead researcher (AA) and a supporting teacher, creating a collaborative and comfortable environment. Data collection included both audio recordings and written observations.

### Piloting the semi-structured interviews and workshops

The topic guides were developed and piloted with two teachers and four male students to assess the clarity, validity, and reliability of the questions. The pilot helped refine the wording, eliminate redundancy, and improve participant comprehension. It also established the average interview duration (35–45 minutes), which informed further adjustments to the guides.

Data from the pilot were excluded from the final dataset to ensure that only validated questions were used.

### Data analysis

Thematic analysis as outlined by Braun and Clarke [22], was used to analyse the data. The lead researcher transcribed interviews and workshop discussions verbatim in Arabic, ensuring accuracy by cross-checking against recordings. As the only bilingual researcher, AA also reviewed the English translations alongside the Arabic transcripts and resolved any uncertainties in meaning through reflexive cross-checking and reference to the original text. Transcripts were imported into MAXQDA-Vision 22 for data organisation and coding [23].

Two transcripts were initially coded by AA, followed by a thorough review by the research team (EG and LB), who provided detailed feedback. This review led to revisions in some of the codes, and the research team finalised the codebook through discussion.

The data were analysed using the SEM framework, categorising themes across individual, interpersonal, organisational, community, and policy levels. Themes were iteratively refined through discussions with the research team, ensuring their relevance and coherence. Reflexivity was maintained throughout the analysis to minimise researcher bias.

### Researcher positionality and reflexivity

The researcher’s background in PE and health sciences, along with being a Saudi national, facilitated participant access and helped shape the study’s focus. However, this shared cultural familiarity also posed potential biases. To mitigate these, strategies such as emphasising voluntary participation, fostering open dialogue, and involving a research assistant were employed.

Reflexivity was maintained throughout the data analysis process to ensure objectivity and enhance the study’s credibility.

### Ethical considerations

Participants provided written informed consent prior to taking part in the study. Consent forms clearly outlined the voluntary nature of participation, confidentiality, and the right to withdraw at any time.

Workshops with students adhered to strict ethical standards, including obtaining both parental consent and assent from the students themselves. A supporting teacher was present to ensure a safe and supportive environment. All data were securely stored on an encrypted server, accessible only to the researcher and the research team.

Additional information regarding the ethical, cultural, and scientific considerations specific to inclusivity in global research is provided in the Supporting Information (S5 File).

### Reliability and validity (trustworthiness)

To ensure the trustworthiness of the qualitative findings, this study addressed reliability and validity through the criteria of dependability, credibility, transferability, and confirmability, following guidelines suggested by [24,25].

Dependability was enhanced through member checking and triangulation techniques [26]. Preliminary findings were shared with participants for feedback and correction. Triangulation involved using multiple data sources and methods, incorporating the perspectives of students, school staff, and parents to corroborate findings and minimise bias [27]. This approach allowed for cross-validation of data, ensuring reliable results through systematic comparison of emerging themes across participant groups.

Credibility was strengthened through prolonged engagement with participants and ongoing reflexivity. Considerable time was invested in building trust and rapport, facilitating honest and detailed disclosures. Reflexivity involved continuous self-reflection on potential biases and their influence on the research process [28].

Transferability was supported by providing detailed descriptions of the research context and methodology, enabling readers to assess the applicability of the findings to other settings [29]. Thick descriptions contributed to a deeper understanding of the participants’ specific environments and experiences.

Confirmability was ensured by maintaining a rigorous audit trail and engaging in peer debriefing with the research team to refine findings and interpretations [29].

## Results

A total of 16 school staff members participated in the study, 9 from a private school and 7 from a public school. Additionally, 37 students (aged 13–15) from both schools took part in two workshops each, and 7 fathers contributed via interviews. The distribution of participants by school is shown in Table 1.

**Table 1:** Characteristics of schools and participants.

Type of School	Private School	Public School
**Location**	Affluent area	Deprived area
**School Staff**	7 teachers, 1 principal, 1 administrator (Total: 9)	5 teachers, 1 principal, 1 coordinator (Total: 7)
**Male Students**	6 aged 13, 7 aged 14, 6 aged 15 (Total: 19)	5 aged 13, 6 aged 14, 7 aged 15 (Total: 18)
**Fathers**	5	2

Initially, 11 private and 9 public school staff members expressed interest in participating. However, 2 teachers from the private school later withdrew, suspecting the researcher was affiliated with the Ministry of Education. In the public school, one teacher declined due to a perceived lack of benefit, and another missed the scheduled interview. Interview durations ranged from 31 to 48 minutes.

Regarding students, 28 from the private school and 24 from the public school initially expressed interest. Ultimately, 19 private and 18 public school students met the inclusion criteria. Nine students did not return parental consent forms for unknown reasons, while six additional students from the public school were unable to participate for the same reason.

Nine fathers initially contacted the researcher to participate. Interview logistics were arranged during phone calls; however, only 7 attended their scheduled interviews.

Findings are structured according to the SEM. Descriptive data about participants and schools are presented first, followed by thematic findings derived through thematic analysis (TA). No new codes emerged after interview 14, indicating that thematic saturation had been achieved at that point.

### Characteristics of the schools and participants

The study involved two schools in Jeddah, KSA, representing contrasting socio-economic environments: a private school in an affluent area and a public school in a deprived area. Sixteen school staff members participated in interviews—nine from the private school and seven from the public school. These staff members ranged in age from 32 to 48 years, with an average of 15 years of teaching experience. All participants were Saudi nationals, and their roles included teaching various subjects, including two who were physical education (PE) teachers.

Thirty-seven male students aged 13–15 participated in the workshops. Nineteen students were from the private school and 18 from the public school. Additionally, seven fathers contributed through interviews. Table 1 summarises the distribution of participants.

### Themes and sub-themes

**Fig 1:**
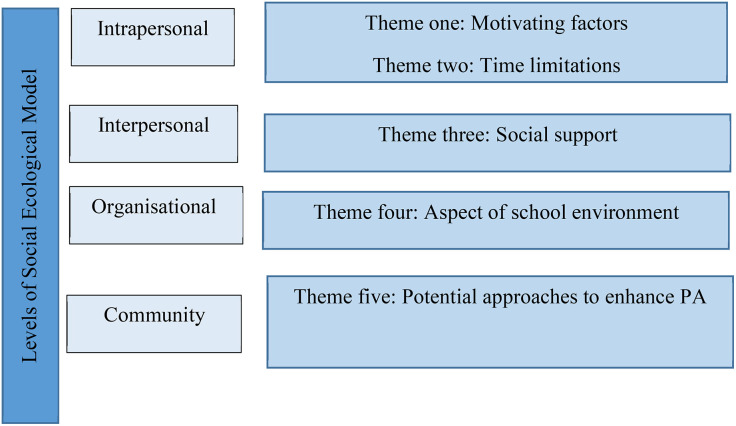
Thematic map of SEM levels and themes.

The findings are structured according to the SEM, highlighting factors influencing PA among male students. Thematic organisation across SEM levels is illustrated in the thematic map (Fig 1), with detailed themes and sub-themes presented in Table 2.

**Table 2:** Themes and sub-themes.

SEM Level	Themes	Sub-themes
Intrapersonal	Motivating Factors	Enjoyment and Competition, Benefits of PA, Behavioral and Psychological Influences
	Time Limitations	Curriculum Overload, Competing Uses of Time
Interpersonal	Social Support	Parental Support, Peer Support, Teacher Support
Organisational	School Environment	Subject Content and Regulations, School PE Class, Inequality in Access to Facilities
Community	Potential Approaches to Enhance PA	Cross-Cultural Adaptation, Local Proposals, Role of Educational Authorities

#### Theme 1: Motivating factors (intrapersonal level).

This theme focuses on the internal factors that influence male students’ engagement in and restriction from PA. Participants expressed that the enjoyment derived from physical activity positively impacted their motivation. Activities perceived as enjoyable—such as team sports or group fitness classes—were more likely to encourage regular participation.

Additionally, competition emerged as a motivating factor for some students, as it fostered a sense of challenge and achievement.

**Enjoyment and competition:** This sub-theme highlights the significant role of enjoyment and competition in motivating students to engage in PA. During workshops and interviews, participants consistently identified personal interest and enjoyment as primary motivators. Teachers from both private and public schools emphasised the importance of competition and personal choice as key drivers of student enthusiasm.

Additionally, many students expressed that having the freedom to select their preferred activities enhanced their overall enjoyment and increased their excitement toward participating in PA.

*“Competition creates enthusiasm among students”.*

Teacher 6, private school.

*“The first thing is the desire to do the activity and [that it will] make me excited while I do it”.*

Student 7, public school.

Several teachers from the private school reported that male students showed greater commitment when engaging in physical activities of their choice, with the added element of competition further boosting their enthusiasm.

*“When students choose the activities they love, their commitment to doing them regularly increases”.*

Teacher 1, private school.

Parents and some teachers also highlighted the importance of allowing children to choose the type of PA they engage in. They noted that such autonomy would significantly encourage participation. One father, for example, stated:

*“The most important point, and [one that] the school should be focusing on it, is to give each student his desire to choose the activity he loves according to his inclinations and desires, because this certainly helps children engage in physical activities”.*

Father 2, private school.

Faith-based motivation emerged as a significant driver of PA engagement. A few teachers from both schools used religious teachings as a means to emphasise the importance of physical strength and activity. The religious perspective was portrayed not only as a way to convey the value of PA, but also as a source of inspiration—encouraging students to view physical strength as a means of fulfilling their religious obligations. This perspective is illustrated by the following example:

*“It has a religious importance in our society: ‘The strong believer is dearer to God than the weak believer.’ I mean physical strength, a legitimate requirement”.*

Teacher 3, private school.

*“PA is significant, and our religion advises us to do activities, and in a hadith from the Prophet, may God’s prayers and peace be upon him. It means that movement is a blessing and that a healthy mind resides in a healthy body”*

(Teacher 2, public school).

The final element was competition. Most male students from both schools expressed enthusiasm for competitive or challenging activities, particularly team sports. Teachers also confirmed the motivational role of competition.

*“The thing we are excited about is that there is competition”.*

Student on Table A, private school.

*“Prizes and competitions. These two things are significant and create enthusiasm among students”*

(Teacher 5, public school).

**The benefits of PA:** This sub-theme explores participants’ perceptions of the benefits of regular PA, highlighting advantages across multiple domains.

The first element focuses on academic benefits. Participants emphasised that engaging in physical activities can positively influence academic performance by increasing concentration, improving comprehension, and enhancing overall achievement. This suggests that PA supports not only physical well-being but also cognitive functioning, contributing to academic success.

The second element addresses the psychological benefits of PA, including stress reduction, mood enhancement, and its potential role in alleviating symptoms of depression.

The third element concerns the physical health benefits of regular PA. Participants recognised that PA helps maintain physical fitness, strengthen muscles, burn fat, and prevent obesity. In addition, it was believed to stimulate the release of ‘happiness hormones,’ improve the circulatory system, and enhance self-image—making these outcomes strong motivators for continued engagement.

The fourth element highlights the mental benefits of PA, such as improved cognitive functions, including memory, attention, and problem-solving skills.

It is worth noting that some participants identified additional benefits of PA, such as weight management and the prevention of obesity. These outcomes were seen as helpful for both avoiding excess weight and supporting weight loss. Furthermore, two teachers shared personal experiences highlighting the health benefits of increased physical activity.

One teacher shared his personal experience, noting that regular walking helped reduce his anxiety and improve his problem-solving abilities.

*“Look, sometimes when problems arise between me and my wife, I immediately get out of the house and go for a walk. Thank God, I discovered during the walk that the anxiety reduces and I begin to think deeply about solving the problem.*

Teacher 8, private school.

**Behavioural and psychological factors influencing PA attitudes:** This sub-theme examines the behavioural and psychological factors that shape students’ attitudes toward PA, highlighting eight key elements. The most prominent factor identified was a lack of skills, which participants viewed as a major barrier to participation. Many male students reported feeling inadequate or unskilled, which discouraged their involvement in PA.

Workshops and interviews revealed that students from the private school more frequently emphasised the need for specialised skills in activities such as volleyball, basketball, and swimming, compared to their public school counterparts. Teachers from both schools also acknowledged that limited proficiency in team sports, particularly football and volleyball, hindered student engagement.

*“We often feel left out in team games because we don’t have the basic skills to compete”*

Student, private school.

*“We, here in the school, have many options in PE such as swimming, volleyball, basketball and tennis, but some students do not have the skills to play enthusiastically”.*

Student, private school.

*“In the PE class, we play football, but some students do not know how to play and the team is incomplete, and thus we sometimes cancel the match”*

(Student, public school).

The second significant barrier identified was a lack of interest in physical activities. Participants explained that when activities did not align with students’ interests, their motivation to engage was greatly reduced. In particular, male students from the public school emphasised the importance of having access to a variety of activities to sustain their enthusiasm and participation in PE.

However, their options were limited, as football was the only sport offered during PE classes. This narrow selection did not cater to all students’ preferences, resulting in decreased engagement. School staff echoed this concern, noting that while many male students preferred sports other than football, the school’s resources were limited to supporting football alone. Participants agreed that the restricted range of activities offered during PE classes failed to reflect the diverse interests of male students, contributing to lower levels of participation.

*“I hate playing football, and the school here only has football, so in PE classes, I sit and watch the students while they play”.*

Student 10, public school.

#### Theme 2: Time limitations (intrapersonal level).

This theme comprises two sub-themes, both of which act as barriers that limit male students’ participation in PA. The first, ‘Curriculum Overload’, addresses the challenges posed by a demanding academic schedule that leaves little time for PA. The second, ‘Competing Uses of Time’, explores students’ difficulties in managing their time effectively to incorporate PA into their daily routines.

**Curriculum overload:** This sub-theme highlights barriers at the individual level that limit male students’ participation in PA due to academic demands. During interviews and workshops, participants were asked to identify obstacles to engaging in PA. The majority of male students, particularly those from the private school, cited increasing academic workload, including study pressure, homework, and exams.

Furthermore, some parents from both schools prioritised academic achievement over PA for their children. In addition, several teachers noted that a heavy curriculum reduced students’ opportunities to participate in PA.

*“The problem is that the crowded curriculum leaves students with no time to engage in PA”.*

Teacher 5 from the private school.

*“Because we are busy with studying and exams, we cannot do PA”.*

Student 6, public school.

**Competing uses of time:** This sub-theme highlights poor time management and the lack of dedicated time for PA in male students’ daily routines, representing a significant barrier to engagement. Nearly all male students reported preferring electronic entertainment and spending considerable time in front of screens, watching television, playing PlayStation, and using smartphones or tablets.

Furthermore, parents expressed dissatisfaction with this pattern:

“*On the other hand, we now have a problem, which is the addiction to electronic games and spending a lot of time in front of the TV. Unfortunately, this did not exist before, and now it is overwhelming”.*

Father 4, private school

*“Today, most children spend most of their time on electronic games, but it may become addictive for some. I mean, for example, my older son gets out of the house a lot and goes with his friends to sports clubs and so on, but my younger son is very attached to electronic games and plays all the time”*

(Father 1, public school).

*“Can we say that the children, in particular, this generation, are preoccupied with things other than PA. This is an important point, frankly, that a large percentage of children are obsessed with electronic games […] and these things take up a very large amount of their time”.*

Father 2, private school.

#### Theme 3: Social support (interpersonal level).

This theme explores the role of support provided by different sources in facilitating PA and how it can influence – positively or negatively – students’ PA. This theme includes three sub-themes: ‘Parental support’ focuses on the influence of parents in encouraging and facilitating PA participation. ‘Peer support’ explores the role of peers/friends and social networks in promoting PA engagement. ‘Teacher support’ examines the significance of teachers’ guidance and encouragement in fostering PA among students.

**Parental support:** This sub-theme focuses on the influence of parents in terms of encouraging their male children to be active and facilitating PA participation. Active male students received support from their parents and family members, which was confirmed by teachers and parents during interviews. This support included encouragement, rewards for exercise and parental modelling. A few male students from the private school rather than from the public school stated they were encouraged by their parents to participate in PA three to four times a week, and it had become a habit. Some students from the public school stated they liked PA when their parents promised them rewards, thus motivating them accordingly. However, several teachers from both schools pointed out that insufficient parental support and a lack of awareness negatively affected children’s involvement in PA, especially when some parents favoured educational achievements over participation in PA for their children.

*“I always encourage my children to exercise, and I always make sure that they exercise at least three times a week”.*

Father 1, private school.

Notably, some male students at the private school emphasised that successful national athletes could serve as role models, potentially inspiring them to participate in PA. One student stated he loved sports because his father was a sportsman and that made him aspire to be like his father. Another student stated he loved to play football because he wanted to be a future Cristiano Ronaldo.

*“There are a lot of things that motivate me to do PA, and the one most important to me is the encouragement from my parents, since my father is a sportsman and he enrolled in a big marathon event a few years ago”.*

Student 1, private school.

**Peer support:** This sub-theme examines the role and influence of friends’ and peers’ support in promoting PA engagement. Many male students from both schools highlighted support from their friends and peers as a strong facilitator of participation in PA, especially in co-participation (playing together) or starting a new activity. This sometimes-involved verbal support. Furthermore, some male students at the private school mentioned that they engaged in PA primarily to socialize and spend time with friends, with some using this time to make new friendships, especially out of school time (neighbourhood friends). However, a few male students in the workshops highlighted that being lonely, having inactive friends, or having friends with other interests negatively influenced their participation in PA.

*“The thing that excites me is the presence of enthusiastic friends, because when one of my friends comes to me and tells me that sports are good, and let’s go for a walk or any activity, I get excited about exercise”*

(Student 6, public school).

Some parents and teachers had the same thoughts about peer support. Teachers at the private school noted that the lack of friends, or having friends who are inactive or have different interests, hindered male students from participating in PA. However, one father mentioned that having friends who have negative interests (for example smoking) kept children away from practicing PA.

*“Of course, as a teacher, I always notice the presence of enthusiastic students, especially in the PE class, who positively influence the rest of their classmates and always enthusiastically encourage the lazy ones and so on”.*

Teacher 2, private school.

**Teacher support:** This sub-theme focuses on the significance of teachers’ guidance and encouraging male students to engage in PA, either by participating with them or encouraging them verbally. Many male students from the private school highlighted the importance of involving PE teachers in the PE session, as they can influence their participation positively, especially when they are supportive and enthusiastic. Some of them justified that by their love for the PE teacher, while some said they could learn from their expertise, especially in activities such as swimming, volleyball and tennis. This sentiment was confirmed by one teacher in the private school, noting that when he participated with students, he could see their obvious motivation, and so his job was to support, encourage and teach them. However, some male students at the public school highlighted that a lack of support from PE teacher during PE sessions impacted negatively on their participation.

*“The presence of the PE teacher with us during PA is very important and makes all of us keen to participate. But if he gives us instructions and leaves, some students sit down and do not participate”.*

Student 4, private school.

In the same context, parents recognised the importance of involving teachers in activities, and they mentioned the significance of encouragement, either tangibly, for example doing the activity, or intangibly, such as through praise or encouragement. Also, they stated that teachers can boost their children’s energy and attentiveness and reduce the amount of time they spend sitting down and not moving in PE sessions. Furthermore, it should be noted that one father suggested linking PA with marks to increase children’s participation in PE. In addition, other parents expressed that their children became excited when doing PA with their parents or a family member, especially if they were offered a financial or physical reward.

*“The participation of the PE teacher with his students during PE classes is very important, as this helps encourage children to participate and increases their enthusiasm instead of sitting”.*

Father 2, public school.

#### Theme 4: Aspect of school environment (organisational level).

This theme focuses on the organisational factors affecting PA participation, and it consists of three sub-themes. ‘Subject content and regulations’ examine how the content and regulations surrounding PE classes can either facilitate or hinder PA engagement. “School PE class’ explores the influence of the school’s PE curriculum and facilities on male students’ access to PA. ‘Inequality in accessing facilities’ addresses the disparities in using PA facilities among different schools.

**Subject content and regulations:** This sub-theme examines how the content and regulations surrounding PE classes can either facilitate or hinder PA engagement. The majority of male students and teachers from both schools highlighted that one session a week was not sufficient (45 minutes). Furthermore, some teachers highlighted that one session a week was scheduled by the Ministry of Education and they had to follow the Ministry’s instructions. Notably, one teacher from the private school argued about the practical time for the session, as it only totalled 30 minutes because 15 minutes were deducted from the session time for male students to change and then return to their class.

*“Students have a weekly class for 45 minutes, but actually I consider it 30 minutes. As we know, students leave for 5 minutes then return to class for 5 minutes and they change their clothes for 5 minutes. All of these are deducted from the actual class time”.*

Teacher 7, private school.

It should be noted that the number of male students in each class is very important since a few teachers highlighted that a huge number of students was considered a barrier to accessing PA facilities. They suggested that 20 or fewer in each class would help in this regard.

*“A large number of students in each class is a barrier since some classes have 40 students or more”.*

Teacher 3, private school.

**School PE class:** This sub-theme examines the influence of the school’s PE curriculum on accessing PA. The PE session is sometimes the only opportunity for male students to engage in PA during school time. Teachers from the public school mentioned some factors limiting involvement, such as limited PA opportunities, lack of a competitive environment and lack of PA diversity. In contrast, teachers from the private school highlighted that the Inequality in Access to Facilities diversity of PA opportunities, availability and accessibility were significant factors in facilitating PA among male students. In addition, many students from the public school highlighted some difficulties with the PE session itself, namely cancellation due to bad weather (too hot) – a sentiment shared by the PE teacher from the public school when he highlighted that hot weather hampered students’ participation in PA. He also stated that it was school policy to safeguard students in hot weather. Conversely, students from the private school did not complain about this issue, since they had an airconditioned playground and could practice PA at any time during the school day, which they considered a strong facilitator.

*“As my colleagues mentioned, the main barrier is the high temperature, because I may get heatstroke”.*

Student 3, public school.

**Inequality in relation to accessing facilities:** This sub-theme addresses the variations in PA facilities in terms of availability and accessibility between the two schools. During the interviews and workshops, the participants were asked to describe the types of PA in each school, how long it lasts and the types of facilities. All male students and teachers in the private school stated that they had one session a week for PA (45 minutes), encompassing multiple activities such as football, volleyball, basketball, tennis, swimming and gym. In addition, the school was airconditioned from the entrance to the exit, and they had three playgrounds; all these features are considered facilitators for PA. Conversely, participants from the public school stated the same time allocated for PA but they had only one playground where students could play only football. Notably, the playground at the public school was of low quality and needed maintenance. Participants from the private school considered the availability and accessibility of facilities, resources, and a good environment as facilitators for PA. Conversely, participants at the public school considered a lack of resources (financial and equipment) and facilities as barriers in this regard. A discussion between a group of students from a public school sitting at table B went as follows:

*“We have football, and it is one session per week. There are no other activities” “Ok, if you have one playground and it is worn out, how can you play other activities?” “[Laughter] I don’t know”.*

Students, public school.

#### Theme 5: Potential approaches to enhance PA (community level).

This final theme explores potential approaches to enhance PA in schools. It comprises three sub-themes that highlight potential interventions to promote PA at the community level. The sub-theme ‘Role of Cross-Cultural Adaptation’ focuses on participants’ views regarding these specific programmes, including their potential adoption in KSA schools and the reasons behind their perspectives. ‘The Role of Local Proposals’ suggests programmes recommended by male children, their parents, and school staff to provide more opportunities for engaging in PA at the school level. The ‘Role of Local Educational Authorities’ underscores the importance of local educational authorities in implementing policies and programmes that encourage PA.

**The role of cross-cultural adaptation:** During interviews and workshops, male participants reviewed various PA interventions implemented locally and internationally, including standing desk initiatives, TDM, and educational programmes. Feedback on these interventions highlighted the importance of cross-cultural adaptation when considering their application in Saudi schools.

The standing desk initiative received mixed reactions. Most students found it unengaging and of little value, though some suggested making participation optional to increase appeal. Teachers raised concerns about financial constraints, lack of support, and limited equipment, though one proposed piloting it in select classes under Ministry of Education supervision.

*“Of course, the programme that you mentioned is beautiful, but to implement such programmes, equipment must be provided, and this equipment will cost the school a lot, so I think it’s too difficult to implement this programme in this school”.*

(Teacher 1, private school).

The TDM programme was well-received by teachers, especially those from the private school with prior experience running similar initiatives. They appreciated its simplicity, minimal resource needs, and potential to strengthen teacher-student relationships. However, barriers such as time constraints, curriculum integration, parental approvals, and climate challenges particularly in Jeddah’s heat were noted. Despite these issues, many students valued the programme’s social aspects and positive influence on teacher-student rapport.

*“We have previous experience regarding the walking programme, which was positive in some way. I mean, during the walking some students felt free to speak about certain issues and it was also good to build great relationships with them rather than in the normal classroom”*

(Teacher 9, private school).

*“The daily walking programme has its disadvantages; for example, the weather is hot and it is difficult to walk in hot weather. If we go outside the school, it is difficult because it is not a safe environment, i.e., a car might hit someone”*

(Teacher 3, public school).

Educational programmes were supported by teachers and parents as a means to raise awareness of PA benefits and health risks associated with inactivity. Some teachers emphasised the need for foundational education before implementing PA initiatives. Students had mixed views: while some appreciated short, informative sessions (10–15 minutes weekly), many preferred practical activities over educational content, citing a lack of competitiveness and engagement in purely educational formats.

*“Let me tell you something. We need an educational programme for students about obesity, its harm, sports, walking and its benefits so that students can be educated on these topics”*

(Teacher 1, public school).

In summary, this sub-theme discovered the role of cross-cultural adaptation and shed light on the advantages and disadvantages of different approaches in this regard. The idea of TDM or a 100-club programme was similar to what has been tried in the private school, and the advantage was that they were easy to implement, as no equipment was required. However, some disadvantages mentioned by the male participants included summer heat and the availability of enough space, especially in the public school. The standing desk programme was disliked by many participants because it requires the financial ability to buy equipment, albeit one teacher suggested it may be worth trying on a sample of classes to explore its feasibility. Finally, educational programmes were liked by teachers and parents and disliked by most students.

**The role of local proposals:** This sub-theme explores PA programmes proposed by male students, parents, and school staff to enhance opportunities for PA at school. Participants described their ideal programmes, specifying preferred timing, frequency, location, facilitators, and rationale.

**Fig 2:**
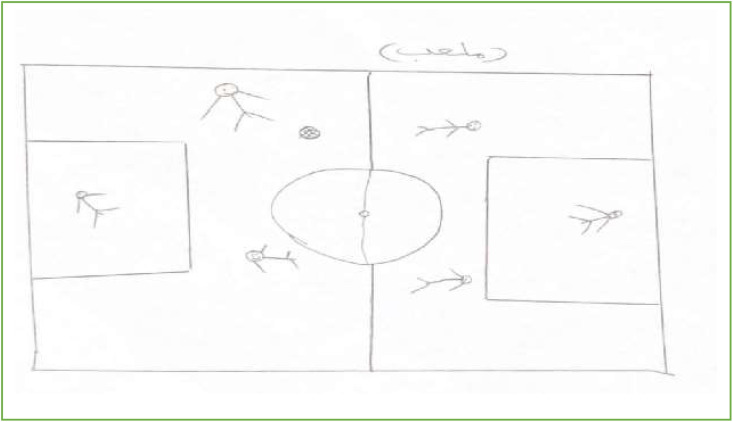
Student group drawing from private school workshop at Table D. This drawing was presented by four male students and shows the playground with students playing football. At the top, the word “playground” is written in Arabic. The drawing reflects the students’ perception that football is the primary physical activity offered during school time, illustrating the limited variety of sports activities available—an issue raised by several participants during workshops.

A strong consensus emerged for offering diverse activities three times a week, led primarily by PE teachers within school premises. Students expressed a desire for variety beyond football, although football three times a week remained a highly favored option due to its popularity and engagement factor (see Fig 2).

**Fig 3:**
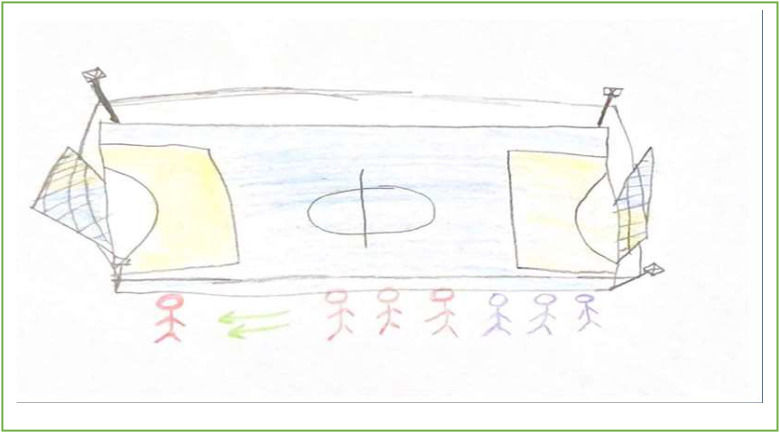
Student group drawing from the public school workshop (Table D). This drawing was presented by four male students and depicts a playground at the centre. The students are shown walking around the playground, following their leader (teacher), who is positioned in front.

The walking programme was the most popular suggestion among all participant groups. Students proposed daily or thrice-weekly sessions, with varying durations (15–45 minutes), generally preferring school staff as facilitators for familiarity and comfort. Teachers and parents supported this idea, emphasising early morning implementation to avoid harsh weather conditions, particularly in the public school with limited facilities (see Fig 3).

*“For me, I chose a daily walking programme, and it does not matter who will apply it, specialists or teachers because it is a simple sport and does not require training”.*

(Student, private school).

Other suggestions included a jogging programme, a weightlifting programme inspired by Swedish approaches, and a step-counting programme that could leverage technology for monitoring and motivation through incentives. These were mostly supported by smaller groups of students and teachers, with an emphasis on staff training prior to implementation.

Additionally, an educational programme combining theoretical knowledge with practical PA was proposed by a few teachers, while one teacher suggested an evening open day programme to offer students more freedom and relaxation outside academic hours.

In summary, this sub-theme highlights the variety of activities suggested by participants from both schools, outlining preferred timings, locations, frequency, and facilitators for these programmes, aimed at increasing opportunities for PA at the school level. The most popular suggestions—namely, a walking programme, various activities three times a week, and football three times a week—received strong support from participants. In contrast, proposals for a jogging programme, weightlifting programme, educational programme, step-counting programme, and an evening programme were made by a smaller group of participants. It was clear from the responses that there is a consensus on the need for three PE sessions per week, with activities preferably conducted inside school premises and led by school staff. Additionally, there was an emphasis on the necessity for proper training of school staff to effectively implement some of these programmes.

**The role of local educational authorities:** This sub-theme highlights the crucial role of local educational authorities in promoting PA within schools through effective policies and programme implementation. During interviews, some parents and teachers emphasised the need for new policies to restructure and enhance school-based PA programmes. Parents from the public school, in particular, voiced dissatisfaction with the limited duration and variety of current physical activities, stressing the importance of providing more time and diverse opportunities for children at this critical developmental stage.

*“There should be new plans, and these plans are supervised and implemented by specialised people from the Ministry. It is useless to have a physical class and tell the students, “Come on, go and play football.”*

(Teacher, public school).

Additionally, participants underscored the broader societal responsibility in encouraging active lifestyles among children. Several parents advocated for increased community awareness and collaboration to foster a culture that values PA. Their input reflects a shared understanding that improving PA in schools requires not only institutional support but also societal engagement.

In conclusion, this theme clarifies the values and priorities of various stakeholders in the context of enhancing PA in KSA school settings. Rather than delineating specific proposals, the findings shed light on the underlying factors deemed important by male students, teachers, and parents. These include the role of cross-cultural adaptation, the role of local proposals, and the role of local educational authorities in implementing such interventions. The responses indicate a broad consensus on the desirability of incorporating PA into school routines, highlighted by eight notable suggestions. Importantly, six of these emerged directly from the students, underscoring their active engagement and interest in shaping PA initiatives. The remaining two suggestions, contributed by teachers and parents, complement this picture by adding an adult perspective to the discussion. This collective input provides valuable insights into what might render specific recommendations more effective and acceptable within the educational environment of KSA.

## Discussion

Utilising a qualitative approach, this study aimed to explore the barriers and enablers related to PA among schoolboys aged 13–15 years in KSA. The findings revealed five key themes, categorised according to the levels of the SEM. This framework was used to facilitate comparison with previous studies, where the SEM has been widely applied.

These barriers and motivators were grouped into five overarching themes, each corresponding to a specific SEM level: two themes at the intrapersonal level (motivating factors and time limitations), one at the interpersonal level (social support from various sources), one at the organisational level (aspects of the school environment), and one at the community level (potential approaches to enhance PA). Some themes encompassed both facilitators and barriers to PA.

Whilst many of these themes reinforce existing research, several findings highlight factors that may be more context-specific or particularly relevant to boys in this age group within Saudi Arabian schools.

### Intrapersonal factors

Motivational factors included enjoyment, competition, perceived benefits of PA, and autonomy in activity choice, all factors identified in previous studies [30,31].

Participants highlighted academic, psychological, and physical benefits which acted as strong motivators. Previous studies also have emphasised the role of perceived competence and positive experiences in promoting PA engagement [31,32].

Conversely, several intrapersonal barriers were identified, such as lack of skills, low interest, excess weight, laziness, fear of injury, and preference for sedentary activities. These are consistent with previous findings in KSA [33] and internationally [34]. Additional barriers included peer bullying, imitation, and excessive screen time, which align with prior research highlighting screen use as a contributor to the global epidemic of inactivity in KSA and globally [35–37].

Academic pressure, exams, and homework, along with poor time management, were significant constraints on PA participation. These findings align with [38], who reported similar barriers among South Asian adolescents. Time constraints as a barrier were also widely reported in other studies [39].

Despite recognition of motivators and barriers, participants demonstrated limited knowledge of PA guidelines and health concepts, consistent with previous studies [40,41]. These findings suggest that awareness alone is insufficient to drive behavior change and highlight the need for strategies addressing both motivational and contextual factors.

Lastly, co-participation, breaking daily routines, and activity choice were strong facilitators, in this study. However, competition, while motivating for boys, may act as a barrier for girls and less skilled individuals [31,42–44], underscoring the importance of gender-sensitive approaches to PA promotion.

### Interpersonal factors

At this level, this study identified one theme that examines the role of support received from parents, peers and teachers.

Social support consistently influenced male students’ engagement in PA, either positively or negatively. This support manifests in diverse ways, ranging from intangible forms such as encouragement, rewards for exercise, and parental modelling to tangible actions like co participation and supervision by school staff.. The findings suggest that boys are particularly influenced by the support and encouragement of their fathers and male teachers, consistent with literature that highlights the role of male role models in promoting PA among boys [45]. In contrast, research involving female participants often emphasises the importance of maternal support and the influence of female role models [46,47]. These gender differences in social support should be considered when designing interventions to ensure they are appropriately targeted and effective.

In the private school, we have identified that social support from various sources, including family, friends/peers, and teachers, has positively influenced students’ engagement in PA. This support provides encouragement, resources, and a positive environment for participation in PA.

Conversely, in the public school, students have concerns about the lack of support as some of them acknowledged that lack of support from friends, teachers, and family members impeded their participation in PA. This lack of support can result in lower motivation and fewer opportunities to engage in PA. While students might not explicitly understand the benefits of support they never received, their feedback indicates that feeling unsupported is a significant barrier to participating in PA. This demonstrates the crucial role that social support plays in influencing students’ engagement in PA. Moreover, the absence of supportive peers or association with friends who engage in negative behaviours, such as smoking or physical inactivity, may further diminish boys PA engagement as mentioned by some parents. Additionally, insufficient support from parents and a lack of awareness had detrimental effects on children’s PA participation, particularly when some parents prioritised educational achievements over PA for their children.

These findings align with a previous qualitative studyamong adolescents to investigate elements that influence both the continuation and decrease of PA during adolescence [32]. They found that adolescents became physically active when they received parental support including participating in sports gatherings and offering support and motivation. Furthermore, they found that lack of support from parents or peers diminished participation in PA.

### Organisational factors

At the organisational level, this study identified one overarching theme: Aspects of the School Environment, comprising three sub-themes: Subject Content and Regulations, School PE Classes, and Inequality in Access to Facilities. Organisational influences are critical in shaping adolescents’ PA engagement, particularly as children often have limited control over their environments [48]. Interventions that address organisational factors, alongside individual and interpersonal levels, have shown greater effectiveness [49].

PE classes were recognised as key opportunities for PA engagement, consistent with findings from Morocco [50]. However, several barriers were identified, including session cancellations due to extreme heat, lack of variety in PA, insufficient equipment, and a shortage of qualified PE teachers. These factors diminished participation, particularly during the summer months when high temperatures limit outdoor activities—a challenge less explored in previous studies [51,52].

Participants also highlighted the limitations of subject content and regulations, noting that one 45-minute PE session per week was inadequate. Additional barriers included time lost to changing clothes and large class sizes, sometimes up to 40 students, reducing students attention and space for activities. These findings are similar to those of [33] and highlight the mismatch between current PE provision and WHO recommendations of at least 60 minutes of daily PA for adolescents [53]. Smaller class sizes have been associated with better engagement and individualised instruction, supporting PA participation [54,55].

Inequality in access to facilities was evident between the two schools studied. The private school in an affluent area provided multiple playgrounds, air-conditioned environments, and diverse PA options such as swimming, tennis, and gym facilities. In contrast, the public school in a deprived area had limited, poorly maintained facilities, restricting students’ PA opportunities to football alone. These disparities, echoed in prior studies [33], illustrate how environmental inequities influence PA engagement.

Participants in the public school expressed dissatisfaction with the limited facilities, equipment, and activity variety, which constrained their PA opportunities. This is consistent with prior reviews showing that access to quality sports equipment and playground conditions are key determinants of children’s PA levels [56]. Additionally, a previous review highlighted the importance of providing opportunities for PA during PE lessons, recess, and active commuting [57].

### Community factors

The final theme was Potential Approaches to Enhance PA, including three sub-themes. The first, The Role of Cross-Cultural Adoption, explored perspectives on adopting PA programmes from Western countries in KSA schools. Participants found some interventions, such as the standing desk programme [58], unsuitable. Students reported it lacked competition or enjoyment, and school staff noted financial constraints and lack of support as barriers. Some teachers viewed standing as punishment, further limiting its acceptance. This perception reflects cultural associations between sitting and attentiveness, similar to findings in workplace settings [59,60]. Despite this, [58] found standing desks reduced BMI and sitting time and increased MVPA and academic performance in other contexts.

TDM was seen as easier to implement but challenged by the summer heat and space limitations in the public school. Similar barriers were reported by [61]. Nonetheless, TDM has been associated with increased MVPA and PF [62], contributing nearly ten additional minutes of MVPA daily. Educational programmes were supported by teachers and parents, but less so by students. While RCTs in Spain found limited PA improvement, systematic review [63] and an umbrella review [16] suggest combined, long-term educational interventions can increase PA and reduce BMI.

The second sub-theme, The Role of Local Proposals, highlighted suggestions from students, parents, and staff. Students from both schools preferred a walking programme, varied activities, and football. While inspired by TDM, they preferred school-led programmes, with suggested durations ranging from daily half-hour sessions to 45-minute sessions three times a week. Yet, as [64] noted, interventions from other regions may not suit GCC contexts due to cultural and climatic factors. Students also requested a variety of activities tailored to different interests and abilities, consistent with findings by [65]. Football was a popular suggestion, as it requires less training and uses existing facilities [33].

The final sub-theme, The Role of Local Educational Authorities, highlighted the need for policy changes to promote PA. Participants stressed increasing PA time beyond the current single 45-minute weekly session. Limited time reduces opportunities for meaningful PA, impacting PF [33]. Adequate PA duration is essential for achieving health benefits [36,50].

### Implications for policy and practice

Based on a comprehensive analysis, recommendations for PA interventions targeting boys in KSA are proposed using the Theory of Expanded, Extended, and Enhanced Opportunities (TEO) [66]. TEO offers a practical framework, developed in response to the limited impact of traditional PA interventions, by focusing on increasing opportunities for PA rather than relying solely on complex behavioural theories.

Beets et al. identified three mechanisms common in effective interventions: expanding opportunities by introducing new occasions for PA, extending existing opportunities by increasing their duration, and enhancing the quality of these opportunities through improved delivery and resources. Applications of the TEO framework in schools and after-school programmes have shown increases in MVPA among youth, with interventions such as after-school activities and more frequent PE classes yielding positive results.

Recent studies further support TEO’s applicability. For example, the Turn Up the HEAT programme in summer camps significantly increased MVPA [67], while a cRCT in Swiss secondary schools demonstrated sustainable increases in PA by applying TEO principles [68]. Additionally, an interdisciplinary obesity prevention programme used TEO to improve PA quality among overweight adolescents before expanding or extending opportunities [69].

For boys in KSA, the TEO framework can guide culturally tailored interventions. Expanded opportunities could include short PA breaks during school hours and after-school programmes offering a range of engaging activities. Extended opportunities may involve increasing recess and PE class durations, with more frequent sessions totalling at least 120 minutes weekly though this recommendation should be explored in larger, representative studies. Enhanced opportunities should focus on diversifying PE curricula to cater to different interests and abilities, professional development for PE teachers, and investment in quality equipment and safe facilities.

### Recommendations for schools

Schools should offer a broad range of PA options, including both competitive and non-competitive activities, to accommodate diverse student preferences and abilities. Incorporating student input into the design of PA programmes can further enhance motivation and engagement. Additionally, public schools should prioritise partnerships with local sports clubs to improve access to facilities and address resource disparities, helping to reduce inequalities in PA opportunities.

### Recommendations for policymakers

Policymakers should mandate more frequent and longer PA sessions within the school curriculum to align with WHO guidelines. National campaigns are also needed to educate parents about the benefits of PA and the risks associated with inactivity, making use of popular social media platforms in KSA to maximise outreach. Additionally, increased investment in infrastructure, equipment, and professional development for PE teachers is essential to ensure the delivery of high-quality PA programmes in schools.

### Strengths and limitations

This study has several strengths that enhance its contribution to the understanding of PA among school-aged boys in Jeddah, KSA. It uniquely gathered perspectives from children, parents, and school staff, offering a comprehensive view of the factors influencing PA within this context. To the best of the researchers’ knowledge, this is the first qualitative study in Jeddah to explore PA facilitators and barriers among boys aged 13–15 using the SEM. The study’s rigorous qualitative approach, including triangulation of perspectives and follow-up interviews, strengthened the reliability and credibility of its findings.

However, the study also has notable limitations. Its focus on boys excludes female students due to cultural barriers, limiting the generalisability of findings across genders. Future studies should include girls to understand gender differences in PA behaviours. The study was conducted in only two intermediate schools in Jeddah, selected from a pool of six schools within the same administrative region. While this allowed for in-depth analysis of two contrasting school environments, the findings may not reflect the broader diversity of school settings across other regions in KSA or among students in different age groups, where other barriers and enablers may exist.

Potential recruitment and social desirability biases could have influenced participants’ responses, and challenges in translating Arabic expressions into English may have affected the interpretation of nuanced meanings. Despite these limitations, the researcher took measures to maintain the integrity of the data, though future studies should consider the impact of translation and cultural context more carefully.

## Conclusion

This study sheds light on the multifaceted factors influencing PA among male adolescents in Jeddah, KSA, offering valuable insights for culturally relevant and context-specific interventions. Addressing the identified barriers and leveraging facilitators can significantly enhance PA engagement, contributing to improved public health outcomes for this population. By prioritising collaborative efforts among schools, policymakers, and communities, sustainable solutions can be implemented to foster an active and healthy youth population in KSA.

## Supporting information

S1 Table Consolidated criteria for reporting qualitative studies (COREQ) checklist. (DOCX)

S1 File Interview guide for school staff. (DOCX)

S2 File Interview guide for parents. (DOCX)

S3 File Workshops guide for students. (DOCX)

S4 File Data. Full set of anonymised participant quotations used to support the thematic analysis presented in the manuscript.
(DOCX)

S5 File Inclusivity in global research checklist. (DOCX)

## References

[1] Alasqah I, Mahmud I, East L, Usher K. Patterns of physical activity and dietary habits among adolescents in Saudi Arabia: a systematic review. Int J Health Sci (Qassim). 2021;15(2):39–48. 33708043 PMC7934132

[2] Hazazi A, Wilson A. Noncommunicable diseases and health system responses in Saudi Arabia: focus on policies and strategies. A qualitative study. Health Res Policy Syst. 2022;20(1):63. doi: 10.1186/s12961-022-00872-9 35698126 PMC9195368

[3] World Health Organization. Physical activity [Internet]. Geneva: WHO; 2023 [cited 2025 Feb 3]. Available from: https://www.who.int/news-room/fact-sheets/detail/physical-activity

[4] Lee IM, Shiroma EJ, Lobelo F, Puska P, Blair SN, Katzmarzyk PT. Effect of physical inactivity on major non-communicable diseases worldwide: an analysis of burden of disease and life expectancy. The Lancet. 2012;380(9838):219–29. 10.1016/S0140-6736(12)61031-9 PMC3645500 22818936

[5] Sharara E, Akik C, Ghattas H, Makhlouf Obermeyer C. Physical inactivity, gender and culture in Arab countries: a systematic assessment of the literature. BMC Public Health. 2018;18(1):639. doi: 10.1186/s12889-018-5472-z 29776343 PMC5960209

[6] Lubans D, Richards J, Hillman C, Faulkner G, Beauchamp M, Nilsson M, et al. Physical activity for cognitive and mental health in youth: a systematic review of mechanisms. Pediatrics. 2016;138(3):e20161642. doi: 10.1542/peds.2016-1642 27542849

[7] Al-Hazzaa HM, AlMarzooqi MA. Descriptive analysis of physical activity initiatives for health promotion in Saudi Arabia. Front Public Health. 2018;6:329. doi: 10.3389/fpubh.2018.00329 30488032 PMC6246731

[8] Alqahtani BA, Alenazi AM, Alhowimel AS, Elnaggar RK. The descriptive pattern of physical activity in Saudi Arabia: analysis of national survey data. Int Health. 2021;13(3):232–9. doi: 10.1093/inthealth/ihaa027 32511710 PMC8079316

[9] Rasberry CN, Lee SM, Robin L, Laris BA, Russell LA, Coyle KK, et al. The association between school-based physical activity, including physical education, and academic performance: a systematic review of the literature. Prev Med. 2011;52 Suppl 1:S10–20. doi: 10.1016/j.ypmed.2011.01.027 21291905

[10] Al-Hazzaa HM, Alahmadi MA, Al-Sobayel HI, Abahussain NA, Qahwaji DM, Musaiger AO. Patterns and determinants of physical activity among Saudi adolescents. J Phys Act Health. 2014;11(6):1202–11. doi: 10.1123/jpah.2012-0427 23963597

[11] Chesham RA, Booth JN, Sweeney EL, Ryde GC, Gorely T, Brooks NE, et al. The Daily Mile makes primary school children more active, less sedentary and improves their fitness and body composition: a quasi-experimental pilot study. BMC Med. 2018;16(1):64. doi: 10.1186/s12916-018-1049-z 29743076 PMC5944120

[12] Masini A, Marini S, Gori D, Leoni E, Rochira A, Dallolio L. Evaluation of school-based interventions of active breaks in primary schools: A systematic review and meta-analysis. J Sci Med Sport. 2020;23(4):377–84. doi: 10.1016/j.jsams.2019.10.008 31722840

[13] Albugami HF. A Physical Activity Intervention to Increase the Level of Physical Fitness Among Boys Aged 14-15 Years in Jeddah City, Saudi Arabia (Doctoral dissertation, University of Technology Sydney (Australia); 2018.

[14] Alalawi A, Blank L, Goyder E. School-based physical activity interventions among children and adolescents in the Middle East and Arabic speaking countries: a systematic review. PLoS One. 2023;18(7):e0288135. doi: 10.1371/journal.pone.0288135 PMC10317243 37399200

[15] Alalawi A, Blank L, Goyder E. Umbrella review of international evidence for the effectiveness of school-based physical activity interventions. PLoS One. 2024;19(6):e0304513. doi: 10.1371/journal.pone.0304513 38870155 PMC11175402

[16] McLeroy KR, Bibeau D, Steckler A, Glanz K. An ecological perspective on health promotion programs. Health Educ Q. 1988;15(4):351–77. doi: 10.1177/109019818801500401 3068205

[17] Denzin NK, Lincoln YS, MacLure M, Otterstad AM, Torrance H, Cannella GS, et al. Critical qualitative methodologies: reconceptualizations and emergent construction. Int Rev Qualit Res. 2017;10(4):482–98.

[18] Tong A, Sainsbury P, Craig J. Consolidated criteria for reporting qualitative research (COREQ): a 32-item checklist for interviews and focus groups. Int J Qual Health Care. 2007;19(6):349–57. doi: 10.1093/intqhc/mzm042 17872937

[19] Daymon C, Holloway I. Qualitative research methods in public relations and marketing communications. London: Routledge; 2010.

[20] Bryman A. Social research methods. Oxford: Oxford University Press; 2016.

[21] Creswell JW, Poth CN. Qualitative inquiry and research design: Choosing among five approaches. Sage publications; 2016.

[22] Braun V, Clarke V. Using thematic analysis in psychology. Qualit Res Psychol. 2006;3(2):77–101.

[23] Kuckartz U, Rädiker S, Kuckartz U, Rädiker S. Introduction: analyzing qualitative data with software. Analyzing qualitative data with MAXQDA: Text, audio, and video. 2019. pp. 1–1.

[24] Pitney WA, Parker J. Qualitative research in physical activity and the health professions. Champaign, IL: Human Kinetics; 2009.

[25] Anderson C. Presenting and evaluating qualitative research. Am J Pharm Educ. 2010;74(8):141. doi: 10.5688/aj7408141 21179252 PMC2987281

[26] Motulsky SL. Is member checking the gold standard of quality in qualitative research? Qualit Psychol. 2021;8(3):389–406. doi: 10.1037/qup0000215

[27] Santiago-Delefosse M, Gavin A, Bruchez C, Roux P, Stephen SL. Quality of qualitative research in the health sciences: Analysis of the common criteria present in 58 assessment guidelines by expert users. Soc Sci Med. 2016;148:142–51. doi: 10.1016/j.socscimed.2015.11.007 26698197

[28] Darawsheh W. Reflexivity in research: promoting rigour, reliability and validity in qualitative research. Int J Ther Rehabil. 2014;21(12):560–8.

[29] Shenton AK. Strategies for ensuring trustworthiness in qualitative research projects. Educ Inform. 2004;22(2):63–75.

[30] Martins J, Marques A, Rodrigues A, Sarmento H, Onofre M, Carreiro da Costa F. Exploring the perspectives of physically active and inactive adolescents: how does physical education influence their lifestyles? Sport Educ Soc. 2016;23(5):505–19. doi: 10.1080/13573322.2016.1229290

[31] Martins J, Marques A, Sarmento H, Carreiro da Costa F. Adolescents’ perspectives on the barriers and facilitators of physical activity: a systematic review of qualitative studies. Health Educ Res. 2015;30(5):742–55. doi: 10.1093/her/cyv042 26324394

[32] Bélanger M, Casey M, Cormier M, Filion AL, Martin G, Aubut S, et al. Maintenance and decline of physical activity during adolescence: insights from a qualitative study. Int J Behav Nutr Phys Act. 2011;8:117. doi: 10.1186/1479-5868-8-117 22017754 PMC3215642

[33] Al-Nuaim A, Safi A. Factors influencing saudi youth physical activity participation: a qualitative study based on the social ecological model. Int J Environ Res Public Health. 2023;20(10):5785. doi: 10.3390/ijerph20105785 37239514 PMC10218016

[34] Koski P, Hirvensalo M, Villberg J, Kokko S. Young people in the social world of physical activities: meanings and barriers. Int J Environ Res Public Health. 2022;19(9):5466. doi: 10.3390/ijerph19095466 35564861 PMC9104600

[35] Badawi S, Farag AA. Young Saudi women’s travel behavior change over 2015/2020. J Transp Health. 2021;21:101080.

[36] Ahmad Bahathig A, Abu Saad H, Md Yusop NB, Mohd Shukri NH, El-Din MME. Relationship between physical activity, sedentary behavior, and anthropometric measurements among saudi female adolescents: a cross-sectional study. Int J Environ Res Public Health. 2021;18(16):8461. doi: 10.3390/ijerph18168461 34444210 PMC8392146

[37] Alahmadi MA, Almasoud KH. Prevalence of sedentary behaviors and sleep duration among Saudi Soccer Players. Int Sci J Phys Educ Sport Sci. 2023;11(1):60–8.

[38] Rajaraman D, Correa N, Punthakee Z, Lear SA, Jayachitra KG, Vaz M, et al. Perceived benefits, facilitators, disadvantages, and barriers for physical activity amongst South Asian Adolescents in India and Canada. J Phys Act Health. 2015;12(7):931–41. doi: 10.1123/jpah.2014-0049 25156451

[39] Safi A, Cole M, Kelly AL, Zariwala MG, Walker NC. Workplace physical activity barriers and facilitators: a qualitative study based on employees physical activity levels. Int J Environ Res Public Health. 2022;19(15):9442. doi: 10.3390/ijerph19159442 35954798 PMC9367711

[40] Marques A, Martins J, Sarmento H, Rocha L, Da Costa FC. Do students know the physical activity recommendations for health promotion? J Phys Act Health. 2015;12(2):253–6. doi: 10.1123/jpah.2013-0228 24769866

[41] Best P, Tully MA, Corepal R, Kee F, Hunter RF. Time to “re-think” physical activity promotion for young people? Results from a repeated cross-sectional study. BMC Public Health. 2017;17(1):208. doi: 10.1186/s12889-017-4136-8 28212634 PMC5316169

[42] Casey MM, Eime RM, Payne WR, Harvey JT. Using a socioecological approach to examine participation in sport and physical activity among rural adolescent girls. Qual Health Res. 2009;19(7):881–93. doi: 10.1177/1049732309338198 19556398

[43] Jonsson L, Berg C, Larsson C, Korp P, Lindgren E-C. Facilitators of Physical Activity: Voices of Adolescents in a Disadvantaged Community. Int J Environ Res Public Health. 2017;14(8):839. doi: 10.3390/ijerph14080839 28933747 PMC5580543

[44] Biddle SJ, Ciaccioni S, Thomas G, Vergeer I. Physical activity and mental health in children and adolescents: an updated review of reviews and an analysis of causality. Psychol Sport Exerc. 2019;42.

[45] Edwardson CL, Gorely T. Parental influences on different types and intensities of physical activity in youth: a systematic review. Psychol Sport Exerc. 2010;11(6):522–35.

[46] Davison KK, Cutting TM, Birch LL. Parents’ activity-related parenting practices predict girls’ physical activity. Med Sci Sports Exerc. 2003;35(9):1589–95. doi: 10.1249/01.MSS.0000084524.19408.0C 12972881 PMC2530913

[47] Pugliese J, Tinsley B. Parental socialization of child and adolescent physical activity: a meta-analysis. J Fam Psychol. 2007;21(3):331–43. doi: 10.1037/0893-3200.21.3.331 17874918

[48] Crespo NC, Corder K, Marshall S, Norman GJ, Patrick K, Sallis JF, et al. An examination of multilevel factors that may explain gender differences in children’s physical activity. J Phys Act Health. 2013;10(7):982–92. doi: 10.1123/jpah.10.7.982 23132842

[49] Heath GW, Parra DC, Sarmiento OL, Andersen LB, Owen N, Goenka S, et al. Evidence-based intervention in physical activity: lessons from around the world. The Lancet. 2012;380(9838):272–81. doi: 10.1016/S0140-6736(12)60816-2 PMC4978123 22818939

[50] Abdelghaffar E-A, Hicham EK, Siham B, Samira EF, Youness EA. Perspectives of adolescents, parents, and teachers on barriers and facilitators of physical activity among school-age adolescents: a qualitative analysis. Environ Health Prev Med. 2019;24(1):21. doi: 10.1186/s12199-019-0775-y 30961543 PMC6454728

[51] Brockman R, Jago R, Fox KR. Children’s active play: self-reported motivators, barriers and facilitators. BMC Public Health. 2011;11:461. doi: 10.1186/1471-2458-11-461 21663605 PMC3124432

[52] Carson V, Spence JC. Seasonal variation in physical activity among children and adolescents: a review. Pediatr Exerc Sci. 2010;22(1):81–92. doi: 10.1123/pes.22.1.81 20332542

[53] World Health Organization (WHO). Global Recommendations on Physical Activity for Health. World Health Organization. [cited 2023 Sep 29]. Available online: https://www.who.int/dietphysicalactivity/factsheet_recommendations/en/

[54] Brabo HC. Class size matters: Impact of class size on differentiating instruction in high school physical education. J Phys Educ. 2023.

[55] Hattie J. The paradox of reducing class size and improving learning outcomes. Int J Educ Res. 2005;43(6):387–425. doi: 10.1016/j.ijer.2006.07.002

[56] Morton KL, Atkin AJ, Corder K, Suhrcke M, van Sluijs EMF. The school environment and adolescent physical activity and sedentary behaviour: a mixed-studies systematic review. Obes Rev. 2016;17(2):142–58. doi: 10.1111/obr.12352 26680609 PMC4914929

[57] Campos‐Garzón P, Sevil‐Serrano J, García‐Hermoso A, Chillón P, Barranco‐Ruiz Y. Contribution of active commuting to and from school to device‐measured physical activity levels in young people: a systematic review and meta‐analysis. Scand J Med Sci Sports. 2023;33(11):2110–24. 37497601 10.1111/sms.14450

[58] Guirado T, Chambonnière C, Chaput J-P, Metz L, Thivel D, Duclos M. Effects of classroom active desks on children and adolescents’ physical activity, sedentary behavior, academic achievements and overall health: a systematic review. Int J Environ Res Public Health. 2021;18(6):2828. doi: 10.3390/ijerph18062828 33802133 PMC7999033

[59] Mackenzie K, Such E, Norman P, Goyder E. Sitting less at work: a qualitative study of barriers and enablers in organisations of different size and sector. BMC Public Health. 2019;19(1):884. doi: 10.1186/s12889-019-7148-8 31272484 PMC6611033

[60] Hall J, Kay T, McConnell A, Mansfield L. “Why would you want to stand?” an account of the lived experience of employees taking part in a workplace sit-stand desk intervention. BMC Public Health. 2019;19(1):1692. doi: 10.1186/s12889-019-8038-9 31847821 PMC6918567

[61] Malden S, Doi L. The Daily Mile: teachers’ perspectives of the barriers and facilitators to the delivery of a school-based physical activity intervention. BMJ Open. 2019;9(3):e027169. doi: 10.1136/bmjopen-2018-027169 30837259 PMC6429867

[62] Breslin G, Hillyard M, Brick N, Shannon S, McKay-Redmond B, McConnell B. A systematic review of the effect of The Daily Mile™ on children’s physical activity, physical health, mental health, wellbeing, academic performance and cognitive function. PLoS One. 2023;18(1):e0277375. doi: 10.1371/journal.pone.0277375 36634113 PMC9836306

[63] Moeini B, Rezapur-Shahkolai F, Bashirian S, Doosti-Irani A, Afshari M, Geravandi A. Effect of interventions based on regular physical activity on weight management in adolescents: a systematic review and a meta-analysis. Syst Rev. 2021;10(1):52. doi: 10.1186/s13643-021-01602-y 33557946 PMC7871535

[64] Pearson F, Huangfu P, Abu-Hijleh FM, Awad SF, Abu-Raddad LJ, Critchley JA. Interventions promoting physical activity among adults and children in the six Gulf Cooperation Council countries: protocol for a systematic review. BMJ Open. 2020;10(8):e037122. doi: 10.1136/bmjopen-2020-037122 32819991 PMC7443261

[65] Casey M, Mooney A, Smyth J, Payne W. Power, regulation and physically active identities: the experiences of rural and regional living adolescent girls. Gender Educ. 2016;28(1):108–27.

[66] Beets MW, Okely A, Weaver RG, Webster C, Lubans D, Brusseau T, et al. The theory of expanded, extended, and enhanced opportunities for youth physical activity promotion. Int J Behav Nutr Phys Act. 2016;13(1):120. doi: 10.1186/s12966-016-0442-2 27852272 PMC5112641

[67] Weaver RG, Brazendale K, Chandler JL, Turner-McGrievy GM, Moore JB, Huberty JL, et al. First year physical activity findings from turn up the HEAT (Healthy Eating and Activity Time) in summer day camps. PLoS One. 2017;12(3):e0173791. doi: 10.1371/journal.pone.0173791 28350830 PMC5369693

[68] Gasser M, Nadenbousch A-M, Egger F, Kamer M, Valkanover S, Schmidt M. Increasing adolescents’ physical activity levels through a comprehensive school-based physical activity program: study protocol of the cluster randomized controlled trial Active School. BMC Pediatr. 2024;24(1):561. doi: 10.1186/s12887-024-05034-0 39232723 PMC11373237

[69] Ten Hoor GA, Plasqui G, Schols AMWJ, Kok G. Development, implementation, and evaluation of an interdisciplinary theory- and evidence-based intervention to prevent childhood obesity: theoretical and methodological lessons learned. Front Public Health. 2017;5:352. doi: 10.3389/fpubh.2017.00352 29312922 PMC5743937

