# Supplementary material for: Exploring barriers and facilitators to physical activity among children in Saudi Arabian schools: A qualitative study
Source: PLoS One. 2025 Sep 15;20(9):e0329600. doi: 10.1371/journal.pone.0329600 (PMC12435728; doi:10.1371/journal.pone.0329600)
Supplement: S1 Table — (DOCX) [file pone.0329600.s001.docx]

**S1 Table. Consolidated criteria for reporting qualitative studies (COREQ) checklist**

| **DOMAIN 1: Research Team and Reflexivity** | | |
| --- | --- | --- |
| **No. Item** | **Guide questions/description** | **Reported on Page #*** |
| 1. **Interviewer/facilitator** | Who conducted the interviews or focus groups? | **7** |
| 1. **Credentials** | What are the researcher's qualifications (e.g., PhD, MSc)? | **phD** |
| 1. **Occupation** | What is the researcher’s professional background? | **7** |
| 1. **Gender** | What is the researcher's gender | **Male** |
| 1. **Experience and training** | What experience or training did the researcher have in qualitative research? | **7** |
| 1. **Relationship established** | Was there a prior relationship between the researcher and participants? | **7** |
| 1. **Participant knowledge of the interviewer** | What did participants know about the researcher (e.g., personal goals, reasons for the research)? | **7** |
| 1. **Interviewer characteristics** | What characteristics of the interviewer (e.g., assumptions, biases) might have influenced the research? | **11 and 12** |
| **DOMAIN 2: Study Design** | | |
| 1. **Sampling method** | How were participants selected (e.g., purposive, convenience)? | **9** |
| 1. **Method of approach** | How were participants approached (e.g., face-to-face, email, phone)? | **9** |
| 1. **Sample size** | How many participants were included? | **9** |
| 1. **Non-participation** | How many refused to participate or dropped out? | **8 and 9.** |
| **Setting** | | |
| 1. **Setting of data collection** | Where were the interviews conducted (e.g., workplace, home, university)? | **6** |
| 1. **Presence of non-participants** | Was anyone else present during the interviews (e.g., family members, colleagues)? | **Only in workshops (teacher assistant)**  **Page 10.** |
| 1. **Description of sample** | What were the key characteristics of the participants (e.g., age, gender, occupation)? | **See table page 13.** |
| **Data Collection** | | |
| 1. **Interview guide** | Were interview questions pilot-tested or reviewed? | **Yes page 11.** |
| 1. **Repeat interviews** | Were interviews repeated with the same participants? | **No** |
| 1. **Audio/visual recording** | Were interviews recorded, and how? | **Yes, pages 9 and 10** |
| 1. **Field notes** | Were field notes used to supplement recordings? | **Yes page 10** |
| 1. **Duration** | What was the average length of interviews or focus groups? | **Page 10.** |
| 1. **Data saturation** | Was data saturation discussed and achieved? | **9** |
| 1. **Transcripts returned** | Were transcripts provided to participants for validation? | **Yes page 11.** |
| **DOMAIN 3: Data Analysis & Reporting**  **Data Analysis** | | |
| 1. **Number of data coders** | How many researchers coded the data? | **11** |
| 1. **Description of the coding** | Was a coding framework used? | **10** |
| 1. **Derivation of themes** | Were themes identified inductively or deductively? | **11** |
| 1. **Software** | What software (if any) was used for analysis (e.g., NVivo)? | MAXQDA-Vision 22, page 10. |
| 1. **Participant checking** | Were participants asked to validate the findings? | **Yes, page 11.** |
| **Reporting** | | |
| 1. **Quotations presented** | Were direct quotes from participants included? | **Yes.** |
| 1. **Data and findings consistency** | Was there consistency between the data presented and the study conclusions? | **Pages 13 to 33.** |
| 1. **Clarity of major themes** | Were major themes clearly defined and supported by data? | **Yes, see Figure 1, page 14.** |
| 1. **Clarity of minor themes** | Were minor themes discussed and contextualized? | **Yes, see pages 13 to 33.** |
| 1. **Reflexivity in reporting** | Did the report reflect on researcher biases or positionality? | **12** |
