# Supplementary material for: Exploring barriers and facilitators to physical activity among children in Saudi Arabian schools: A qualitative study
Source: PLoS One. 2025 Sep 15;20(9):e0329600. doi: 10.1371/journal.pone.0329600 (PMC12435728; doi:10.1371/journal.pone.0329600)
Supplement: S1 File — (DOCX) [file pone.0329600.s002.docx]

**S1 File. Interview guide for school staff**

| 1. | What is your view of PA in daily life? Is it something that’s important to you? |
| --- | --- |
| 2. | What types of physical activities are presently conducted in this school? Please tell me about it, and how long does it last? |
| 3. | Does the current PA in your school educate, or is it sufficient to increase student’s PA levels? |
| 4. | In your school, do you have any type of event that encourages students to be more physically active? If yes, can you tell me more about it? |
| 5. | What do you think are the advantages and disadvantages of the different approaches that have been implemented in Arabic countries and globally, as we have discussed? Do you think some of them would be applicable in your school? Which one? Why do you think that? |
| 6. | How can we help students to be more active and motivated to participate in PA in your school? Please expand on your answer. |
| 7. | If you were offered to have a PA program in your school, how would you go about it? Is there a school policy for PA? |
| 8. | What do you think would be the best time to implement a PA programme? inside a school or after school? Where? How often? By whom, school staff or people from outside? Why do you think that? |
| 9. | What are the barriers to implementing a PA programme in your school? How would you overcome these barriers? |
| 10 | Are there any other points you would like to discuss in this regard? |
