# Supplementary material for: Exploring barriers and facilitators to physical activity among children in Saudi Arabian schools: A qualitative study
Source: PLoS One. 2025 Sep 15;20(9):e0329600. doi: 10.1371/journal.pone.0329600 (PMC12435728; doi:10.1371/journal.pone.0329600)
Supplement: S2 File — (DOCX) [file pone.0329600.s003.docx]

**S2 File. Interview guide for parents**

| What is your view of PA in daily life? Is it something that’s important to you? | .1 |
| --- | --- |
| In your view, what are the benefits of participating in regular physical activities for children? | .2 |
| During your childhood in school, how physically active were you? When you were at school, do you think you had more, or fewer opportunities compared to what your son has nowadays either in school time or in your neighborhoods? | .3 |
| What do you think are the barriers to participating in PA for school children in school time? | .4 |
| In your view, do you think PA is very important as other academic subjects in school for students or not? Why do you think that? | .5 |
| Can you identify the best way to inform or advise schoolchildren about the importance of PA? | .6 |
| Do you have any ideas on ways to improve or encourage schoolchildren to promote PA in schools? | .7 |
| If you had the opportunity to choose a PA programme for school children, what would you choose for them? When? In school time or after school? By whom? School teachers or professionals from outside? How many times? Why do you think that? | .8 |
| In your opinion, what are the obstacles that limit the practice of PA in schools? Why do you think that? | .9 |
| What do you think would help and make students motivated to participate in PA? | .10 |
| Are there any other points you would like to discuss in this regard? | .11 |
