# Supplementary material for: Exploring barriers and facilitators to physical activity among children in Saudi Arabian schools: A qualitative study
Source: PLoS One. 2025 Sep 15;20(9):e0329600. doi: 10.1371/journal.pone.0329600 (PMC12435728; doi:10.1371/journal.pone.0329600)
Supplement: S3 File — (DOCX) [file pone.0329600.s004.docx]

**S3 File. Workshops guide for students**

| .1 | What is your view of PA in daily life? Is it something that’s important to you? |
| --- | --- |
| .2 | In your view, what are the benefits of participating in regular physical activities? |
| .3 | What types of PA do you have in this school? |
| .4 | What are the barriers to participating in PA? |
| .5 | What do you think are the advantages and disadvantages of the different approaches that have been implemented in Arabic countries and globally, as we have discussed? Do you think some of them would be applicable in your school? Which one? Why do you think that? |
| .6 | What do you think would help you and make you motivated to participate in PA? |
| .7 | If we were to offer you an opportunity to choose whatever you like to do as PA, what would you suggest doing? Within the school or after school time? When? How often? For how long? Why do you think that? |
| .8 | How would you like to do PA? By whom, school staff or people from outside the school? Why do you think that? |
| .9 | Are there any other points you would like to discuss in this regard? |
