# Supplementary material for: Exploring barriers and facilitators to physical activity among children in Saudi Arabian schools: A qualitative study
Source: PLoS One. 2025 Sep 15;20(9):e0329600. doi: 10.1371/journal.pone.0329600 (PMC12435728; doi:10.1371/journal.pone.0329600)
Supplement: S4 File — Full set of anonymised participant quotations used to support the thematic analysis presented in the manuscript. (DOCX) [file pone.0329600.s005.docx]

**S4 File. Data. Full set of anonymised participant quotations used to support the thematic analysis presented in the manuscript.**

“Competition creates enthusiasm among students”

(Teacher 6, private school).

“The first thing is the desire to do the activity and [that it will] make me excited while I do it”

(Student 7, public school).

“When students choose the activities they love, their commitment to doing them regularly increases. Adding a competitive element then fuels their enthusiasm even more”

(Teacher 1, private school).

“It is very, very important that I allow the students to practise the PA that they like. Your hand fingers are not the same, meaning that there is a student who loves soccer and another student who loves swimming, and so on”

(Teacher 5, private school).

“The most important point, and [one that] the school should be focusing on it, is to give each student his desire to choose the activity he loves according to his inclinations and desires, because this certainly helps children engage in physical activities”

(Father 2, private school).

“What excites students most is the desire and freedom of choice, and each student has special inclinations: some of them love soccer, some like to practise walking only and some of them like swimming. Therefore, it is necessary to give the student his choice and not force him to engage in a particular activity”

(Teacher 3, private school).

“It has a religious importance in our society: ‘The strong believer is dearer to God than the weak believer.’ I mean physical strength, a legitimate requirement”

(Teacher 3, private school).

“PA is significant, and our religion advises us to do activities, and in a hadith from the Prophet, may God’s prayers and peace be upon him. It means that movement is a blessing and that a healthy mind resides in a healthy body”

(Teacher 2, public school).

“The thing we are excited about is that there is competition”

(Student on Table A, private school)

“Competition creates enthusiasm among students”

(Teacher 6, private school).

“Prizes and competitions. These two things are significant and create enthusiasm among students”

(Teacher 5, public school).

“When we walk, we are active and excited to absorb the lessons because walking activity activates our memory and keeps us focused on the lessons”

(Student on Table B, private school).

“You find him energetic, clear-minded and able to absorb lessons”

(Teacher 4, public school).

“I notice students who engage in PA demonstrate better focus and understanding in their academic work”

(Teacher 8, private school).

“Also, on the level of the student’s performance – I mean, let me give you an example: if the school day begins with a class of sports activity, this will have a positive impact on the student in terms of his understanding of the lessons”

(Father, public school).

“Walking relieves stress, and I believe that it is one of the best ways to get rid of loneliness, especially since I am single and live by myself. I get used to walking for half an hour to 40 minutes each day, and I find myself relieved from loneliness and return home in a good mood, so I think PA very, very important for everyone”

(Teacher 6, private school).

“I tried with my son. Sometimes, we walk in the early morning for nearly half an hour before he goes to school. By God, I have discovered that my son’s mood improves a hundred percent and he goes to school with a clear mind, as well as happy and active.”

(Father 5, private school).

“For me, it is an important thing, and sometimes, when I go with my parents for a walk, I feel energetic and I go back home to study in a good mood and full of motivation”

(Student 13, private school).

“PA is important because it activates the mind, improves mood and reduces stress. We have noticed that, especially when the exercise class is in the early morning, we feel active for the rest of the classes”

(Student, private school).

“On the day when we have a physical class, we feel we are active and in a good mood, but on the days when we do not have a physical class, we feel bored and lethargic because we want to move and do sports”

(Student on Table C, public school).

“PA is significant because it provides physical fitness, and for those who are getting older, their body will be healthy and fit”

(Student on Table D, private school).

“PA maintains physical fitness and body health and protects you, after God Almighty, from diseases”

(Teacher 5, public school).

“It also strengthens the muscles of the body and the muscles of the heart and makes the body continuously active”

(Student on Table A, private school).

“PA is important because it helps build the body and strengthen the muscles.”

(Students on Table C, public school).

“I have a personal experience. I had an increase in triglycerides, and it reached a high level of 500 or more. One of the [things I had to do] was to adhere to a daily programme for a period of six months. The programme consisted of brisk walking each day for 45 minutes and sometimes cycling at home. Thank God, I lost weight and my psychological state improved greatly, and the tests proved that the triglyceride level returned to the normal rate, less than 200. Therefore, I can attest that PA is extremely important”

(Teacher 3, Private School).

“I was overweight at one point, with my weight nearing 90 kilograms. Thanks to God Almighty, and through exercising and walking, my weight dropped to 49 kilograms, without undergoing any surgical operation or anything else—just by brisk walking daily. That is why I highly commend the importance of sports”

(Teacher 5, public school).

“In my opinion, PA is crucial because it prevents obesity and is very beneficial for burning fat”

(Student 15, private school).

“We do PA to care about our body and to avoid being obese”

(Student on Table B, private school).

“Look, sometimes when problems arise between me and my wife, I immediately get out of the house and go for a walk. Thank God, I discovered during the walk that the anxiety reduces and I begin to think deeply about solving the problem. Therefore, I very much praise the importance of sports, specifically walking”

(Teacher 8, private school).

“We often feel left out in team games because we don’t have the basic skills to compete”

(Student, private school).

“We, here in the school, have many options in PE such as swimming, volleyball, basketball and tennis, but some students do not have the skills to play enthusiastically”

(Student, private school).

“In the PE class, we play football, but some students do not know how to play and the team is incomplete, and thus we sometimes cancel the match”

(Student, public school).

“I hate playing football, and the school here only has football, so in PE classes, I sit and watch the students while they play”

(Student 10, public school).

“As a teacher, if I had many activities and options in this school, I would gather all the students, but I have only football and some students do not like that. So, what can I do in this case?”

(Teacher 6, public school).

“Many students feel uncomfortable participating in school activities due to their extra weight and fear of bullying”

(Teacher 5, public school).

“Many students shy away from physical activities due to the fear of being bullied, particularly those who are overweight. The onus is on us, as teachers, to manage this”

(Teacher 4, private school).

“Older students often interrupt our games, demanding to join or they won’t let us play”

(Student on Table A, public school).

“I usually spend my day playing PlayStation and doing homework. I hardly find time for PA”

(Student 13, private school).

“In this era, most children are engrossed in mobile phones, social media and electronic games, [thus] drastically reducing their PA”

(Father 3, private school).

"Sometimes, it's not that we don't want to be active; it's just easier to do nothing when you're not already in the habit of being active."

(Student 6, private school).

“The first reason is laziness. For example, when I organise my time and schedule half an hour for exercise, I lie in bed, and when the time comes, I feel lazy and do not go”

(Student 11, private school).

“My enthusiasm for playing electronic games often results in me feeling lazy and unwilling to engage in physical activities” (Student 3, private school). “The thrill of progressing in my games leaves me too drained and lazy to do any PA”

(Student 8, public school).

“Four years ago, during PE class […] one of our students got injured and we called an ambulance. Later, it turned out that his leg had broken [the] student didn’t play soccer for three years because he was afraid of another injury, even though he was [very proficient] at soccer; therefore, these days, many students fear from injury”

(Teacher 9, private school).

“I avoid physical activities because I’m scared of getting hurt” (Student 15, public school). “Some students shy away from physical activities because they’re afraid of injuries”

(Teacher 4, private school).

“On a personal level, by God, I suffer with my son. Sometimes, I tell him, let's go to the walkway. He says no, I will go with my friends. Let's go to the stadium, he says no, I will go with my friends. I mean, if his friends play, he plays, and if they want to play on the PlayStation, he goes with them…”

(Father 4, private school).

“Sometimes, we are swayed by our friends. If they aren’t interested in participating, we end up following their lead”

(Student on Table D, private school).

“Some of the children, through my discussions with them, do what their peers do, such as sitting for a long time at home and being preoccupied with electronic games. This takes up a large amount of their time and is considered very negative. It should be paid attention to by researchers or specialists, and a solution to this problem should be found”

(Father 5, private school).

“One of the barriers I see is the pressure of study and a large number of assignments and exams”

(Student14, private school).

“The problem is that the crowded curriculum leaves students with no time to engage in PA”

(Teacher 5, private school).

“Because we are busy with studying and exams, we cannot do PA”

(Student 6, public school).

“The pressure of studying and exams, and when I want to go to practise any activity, my father tells me to go and take care of my lessons”.

(Student 12, public school).

“The second obstacle, in my view, is the presence of academic and home pressures on children, such as exams and homework”

(Father 2, public school).

“On the other hand, we now have a problem, which is the addiction to electronic games and spending a lot of time in front of the TV. Unfortunately, this did not exist before, and now it is overwhelming”

(Father 4, private school).

“Can we say that the children, in particular, this generation, are preoccupied with things other than PA. This is an important point, frankly, that a large percentage of children are obsessed with electronic games […] and these things take up a very large amount of their time”

(Father 2, private school).

“Today, most children spend most of their time on electronic games, but it may become addictive for some. I mean, for example, my older son gets out of the house a lot and goes with his friends to sports clubs and so on, but my younger son is very attached to electronic games and plays all the time”

(Father 1, public school).

“For example, how can I allocate time for PA while I am at school all day long, and then, when I go home, I sleep in the afternoon and after that I do my homework and study”

(Student 8, public school).

“I always encourage my children to exercise, and I always make sure that they exercise at least three times a week”

(Father 1, private school).

“My father always motivates me and encourages me to participate in sports activities, and the best thing I love is when he gives me a reward for my participation in sports”

(Student 7, private school).

“Unfortunately, some parents do not cooperate with the school in this aspect. Rather, some of them consider the sports session a waste of time, and they’re indifferent to the importance of sports and the many benefits they bring. Let me give you an example: sometimes, the school holds evening activities and we invite all students to come and choose the activity that suits them, because the school is large, as you can see, and it has many resources, but on the other hand, we see that parents' cooperation is weak in responding to such activities”

(Teacher 4, private school).

“There are a lot of things that motivate me to do PA, and the one most important to me is the encouragement from my parents, since my father is a sportsman and he enrolled in a big marathon event a few years ago”

(Student 1, private school).

“I am the one who loves football and I play every day with my parents and friends’ encouragement because I want to be like Cristiano Ronaldo in the future. Ask my friends, I am the best player in the school”

(Student 6, private school).

“The second thing that motivates me is the presence of friends, because when I exercise alone, I feel bored”

(Student 15, private school).

“To be honest, when we have companionship, and the more the number increases, the more fun and enthusiasm, and especially while we engage in any activity, we chat with each other and laugh. I love team activities”

(Student 12, private school).

“The thing that excites me is the presence of enthusiastic friends, because when one of my friends comes to me and tells me that sports are good, and let's go for a walk or any activity, I get excited about exercise”

(Student 6, public school).

“Of course, as a teacher, I always notice the presence of enthusiastic students, especially in the PE class, who positively influence the rest of their classmates and always enthusiastically encourage the lazy ones and so on”

(Teacher 2, private school).

“I swear, children are always influenced by their peers. I mean, for example, now, if I give advice to my son and his classmate gives him advice, for sure he will take more notice of his colleague than me because they are adolescents. The same thing applies to PA. The boy who loves sports certainly encourages his colleagues to do so. And the lazy one will encourage the rest to be lazy. Also, some students have bad habits, such as smoking, while for sure will keep adolescents away from PA”

(Father 3, private school).

“The presence of the PE teacher with us during PA is very important and makes all of us keen to participate. But if he gives us instructions and leaves, some students sit down and do not participate”

(Student 4, private school).

“We love the participation of the PE teacher with us in class, especially if it is swimming or volleyball because he teaches us some skills”

(Student on Table C, private school).

“As a teacher, I love all of my students and consider them my children. I am always keen to participate in physical activities with them because I have noticed that their enthusiasm increases and they want to do so. So, I am here for them, supporting them and educating them – and that is my duty”

(Teacher, private school).

“The participation of the PE teacher with his students during PE classes is very important, as this helps encourage children to participate and increases their enthusiasm instead of sitting”

(Father 2, public school).

“If the PE teacher is keen to participate with his students in sports activities, this will have a direct impact on the participation of all students. I also suggest that if participation were linked to physical activities to certain degrees, for sure this would motivate students to participate”

(Father 1, private school).

“Previously, I have noticed when I encourage my son to get involved in any activity, or when I give him a reward for his participation in any PA, he gets excited about it. Therefore, the element of reward or encouragement for children is very important, especially at this age”

(Father 3, private school).

“Students have a weekly class for 45 minutes, but actually I consider it 30 minutes. As we know, students leave for 5 minutes then return to class for 5 minutes and they change their clothes for 5 minutes. All of these are deducted from the actual class time”

(Teacher 7, private school).

“There is a specific time for PA, which is a weekly class. The school is here for education, and the most important thing is the educational process and giving classes. As for it being only a weekly class for sports, this is a schedule that comes from the Ministry, and we must follow it”

(Teacher 7, private school).

“A large number of students in each class is a barrier since some classes have 40 students or more”

(Teacher 3, private school).

“Also, as a third obstacle, we have a large number of students in classes, meaning that some have 40 students and the PE class is 45 minutes, meaning an average of one minute for each student. How is it possible for a PE teacher to make all students participate in the activity? The number of students in classes must be fewer than 20”

(Teacher 4, private school).

“As my colleagues mentioned, the main barrier is the high temperature, because I may get heatstroke”

(Student 3, public school).

“As you know, the weather here is very hot, especially in the summer, and that may reduce students’ participation in physical activities on a regular basis”

(Teacher 2, public school).

“If you go outside the walls of the school, you will find the situation very difficult and it is impossible to do any PA, even if we say after 9 am inside the school, it will be very difficult. I swear to God, the weather is hell, and the middle school students are teenagers and young children. From my point of view, they cannot bear the heat of the air. Also, those students are under our responsibility, and it is difficult to allow them to engage in any activity in the hot weather”

(Teacher 4, public school).

“The school here, praise be to God, is fully air-conditioned from entry to exit. Even the gymnasiums and playgrounds have been adapted, so sometimes the sports class is at 10 or 11 in the morning and we do not face any problems because the atmosphere is ready for students to practice any activity. This is something you will not see in other schools in Jeddah”

(Teacher 2, private school).

“We have football, and it is one session per week. There are no other activities” “Ok, if you have one playground and it is worn out, how can you play other activities?” “[Laughter] I don’t know”

(Students, public school).

“We have a range of activities in our school for students, such as football, basketball, volleyball and swimming. In addition to that, we have other games such as Ferrara, as well as artificial intelligence games such as Robot. These are games that develop thinking skills and help students develop their ideas”

(Teacher 4, private school).

“The unavailability of space is the first obstacle, and the second obstacle is the financial situation. In order to overcome these obstacles, I suggest that all teachers cooperate and collect a sum of money from everyone, and we maintain the playground to be suitable standard for students”

(Teacher 2, public school).

“Unfortunately, we do not have suitable facilities for any activities other than football. We have one playground and, unfortunately, it is not well maintained – you can see it if you want”.

(Teacher 4, public school).

“School is supposed to provide various and different physical activities, because sometimes my son at home has little movement and the home environment does not encourage him to do activities, but the school where he spends a long time should provide different physical activities for the children”

(Father 2, public school).

“It does not have any equipment other than for football. In football, in fact, the light student is the one who participates and plays, while the fat one is either a goalkeeper or watches. He is deprived and poor, he himself plays, but he is not able to do so. The Ministry must provide the appropriate facilities and equipment for each school, as well as provide playgrounds for activities. These children are the pleasure of our lives, and they deserve every beautiful thing from us”

(Father, public school).

“The programme in which the student is standing next to the desks I didn’t like it and I think it is not appropriate” “Yeah, I agree with you, and I believe there is no motivation to do that” “You are lazy [laughing], it’s a good programme for lazy people” “For me, I think it’s difficult to stand during lessons, and I don’t think there are any benefits to doing that” “Better to keep it optional for use. If we want to stand, then okay, but if not, we will sit”

(Students on Table B, private school).

“Of course, the programme that you mentioned is beautiful, but to implement such programmes, equipment must be provided, and this equipment will cost the school a lot, so I think it’s too difficult to implement this programme in this school”

(Teacher 1, private school).

“I do not think that this programme is suitable for students, especially at this stage. It may be suitable for high school students or university students but not for teenagers. However, there is no objection to conducting an experimental study in some classes to see the extent of its usefulness. It is better for the Ministry to adopt the experience of such programmes”

(Teacher 9, private school).

“The walking programme is very useful, especially as some students come to school early in the morning and can be sleepy. If we apply this programme in the early morning before classes, all students will be active, and it is better to apply it every day from half an hour to 40 minutes”

(Teacher 6, private school).

“We have previous experience regarding the walking programme, which was positive in some way. I mean, during the walking some students felt free to speak about certain issues and it was also good to build great relationships with them rather than in the normal classroom”

(Teacher 9, private school).

“The walking programme is very beautiful and has many advantages and disadvantages as well. One of its disadvantages is that there is a great responsibility on the teacher when taking all the students out of the school, because any student who gets into trouble is the teacher’s responsibility, and in order to implement a programme like this, we must reduce the time of classes and thus reduce educational attainment”

(Teacher 1, public school).

“The daily walking programme has its disadvantages; for example, the weather is hot and it is difficult to walk in hot weather. If we go outside the school, it is difficult because it is not a safe environment, i.e., a car might hit someone”

(Teacher 3, public school).

“Honestly, I will give you my opinion. I see this programme as excellent and easy to do, and I think it’s applicable in our school because, as I mentioned to you, I always walk with my father and I see the benefits of walking by myself, so I see the walking programme as nice and applicable. I mean, the space in the school is not that large, but it is possible for every class to walk at a certain time”

(Student 4, public school).

“I agree with my colleague that the walking programme is good, because it is a group practice, meaning that we can all walk and talk with each other, and if the teacher walks with us, it is also better because the students get excited and something good happens for once”

(Student 5, public school).

“The daily walking programme is nice, but there should be a prepared place for its implementation, for example a large yard, and it would be better if it was air-conditioned so that it would be easy for us to implement it, because as you know here the weather is sometimes hot, especially in the summer. As such, it would be difficult to implement it outside the school or, for example, inside the school after the third class in the morning. It may be easy to apply in European countries, because I always see in movies that their weather is cold and they have rain, and this thing definitely makes them excited to walk”

(Student 7, public school).

“Also, before the Corona pandemic, we applied a specific programme called the ‘Obesity Program’, and I was responsible for implementing it. It was informative and educated about obesity, the harm it can cause and the factors that lead to it, such as soft drinks, etc. This programme was implemented for one week for all intermediate school classes, and it was useful but quite short, I mean for one week”

(Teacher 1, private school).

“We also have some students who do not know the meaning of physical fitness and do not know how to measure heartbeats and what these indicators can tell us” (Teacher 1, private school). “Let me tell you something. We need an educational programme for students about obesity, its harm, sports, walking and its benefits so that students can be educated on these topics”

(Teacher 1, public school).

“I suggest that we add an additional class to educate students about the importance of activity and give them examples of the most important health benefits as a result of regular PA”

(Teacher 6, private school).

“Educational programmes can be useful if, for example, the class lasts for the first ten minutes of teaching or educating about the importance of walking and sports and then we go to the playground and practice PA”

(Student 16, private school).

“[..] Today if we want to learn something, we can read it on the Internet or watch YouTube”

(Student, public school).

“My own opinion is that the PE class is supposed to be on a daily basis”.

(Teacher 9, private school).

“In my view, it is not enough, and students need two or three classes per week”.

(Teacher 3, private school).

“The student in the intermediate school has tremendous capabilities and must unload them. What I knew from my son, there is only one activity which is football, so I see that there should be at least two to three classes per week for practising sports”.

(Father, public school).

“For me, I chose a daily walking programme, and it does not matter who will apply it, specialists or teachers because it is a simple sport and does not require training”.

(Student, private school).

“It is best to be applied by the school staff because it is difficult to supervise our children by someone from outside the school, even if they specialised in PA”.

(Father, public school).

“If I had the opportunity, I would choose a walking programme for the students, and the best, in my opinion, is that it be three times a week, and it is a full session, which means 45 minutes. Of course, it is better for it to be applied by us as teachers, because as you know, walking is very easy and does not need specialists, as well as it is useful for everyone”.

(Teacher 3, private school).

“Look, no matter how many programmes there are, football remains the favourite game and the popular game for most students”.

(Teacher 9, private school).

“If I were offered the opportunity, immediately, I would choose physical fitness exercise, “a Swedish programme”, which is very important, especially for adolescents, because the physical loads of our children are almost zero. They do not have any physical stamina, and therefore I think that these exercises should be mandatory and not optional”.

(Father 3, private school).

“I see that there is a need for PA with education. I mean, there should be an educational programme about walking and its benefits and so on. At the same time, we apply the walking programme, which means 10% of the class time is education, and then walking practice”.

(Teacher 6, private school).

“If I have the opportunity, I will choose an educational programme for weight loss for students who have obesity, and I can combine it with another programme so that means I educate students first and then allow them to practice”.

(Teacher 5, public school).

“Best applied during school time and two to three times a week. See if the schoolteachers are fully aware of the programme and are able to apply it, that’s ok. but if they do not have sufficient training, we can seek help from specialists from outside the school”.

(Teacher 5, public school).

“All the programmes and examples that you mentioned are very beautiful in my opinion, but they may not suit us due to different customs and traditions. But why we don't choose a programme by ourselves, for example, there is a programme, which is a pedometer from Apple, we can use such a programme to measure the student's step counter for each week and reward the student who achieves the goal”.

(Teacher 1, private school).

“My idea is to have an app for the school to measure the number of steps, movement, and stair climbing, and it will be linked to a database so that we know the level of PA for each student. In the sense that you have data on which to base your choice of the programme”.

(Teacher 6, private school).

“It is best to be applied by school teachers because students accept their teachers more than a stranger and the response rate will be very high but teachers should get enough training before the implementation”.

(Teacher 1, private school).

“If you leave the choice to me, I will choose an evening programme for the students, so that the students attend and practise all the activities they want. Why in the evening, because in the evening we have a long time and away from the pressures of school and the pressures of studying and such things. Why did I choose this programme? Because I saw students in another school attending in the evening on a weekly basis, and I noticed that it was a very wonderful social work, and the students were eager to participate”.

(Teacher 9, private school).

“Yes, regarding the PA, I wish the societal discussion was more serious and broader. Everyone surrounding the children must be involved, i.e. the imam of the mosque must talk about the importance of PA, as well as social media celebrities, celebrities of sports activities, and talk shows on television. I think we need this kind of attention and cooperation. They must focus on an important point, which is how to encourage children in schools to be more active.

(Father, private school).

“There should be new plans, and these plans are supervised and implemented by specialised people from the Ministry. It is useless to have a physical class and tell the students, “Come on, go and play football.”

(Teacher, public school).

“However, I advise all schools not to be limited to one PA type, but to diversify it. Students, especially at this age, have a great need to practice physical activities because this age stage is the basis for building. If the student gets used to practising activities at this age, he will continue and become a habit even when he grows up. Therefore, I see that PA, especially at this age, is very, very important”.

(Father, public school).

“If you notice, most of the students here are from poor neighbourhoods, and some of them cannot register in a sports club and pay 200 to 300 riyals as a monthly subscription fee. Therefore, the Ministry and local authorities must do their best to enact new policies for PA”.

(Teacher, public school).


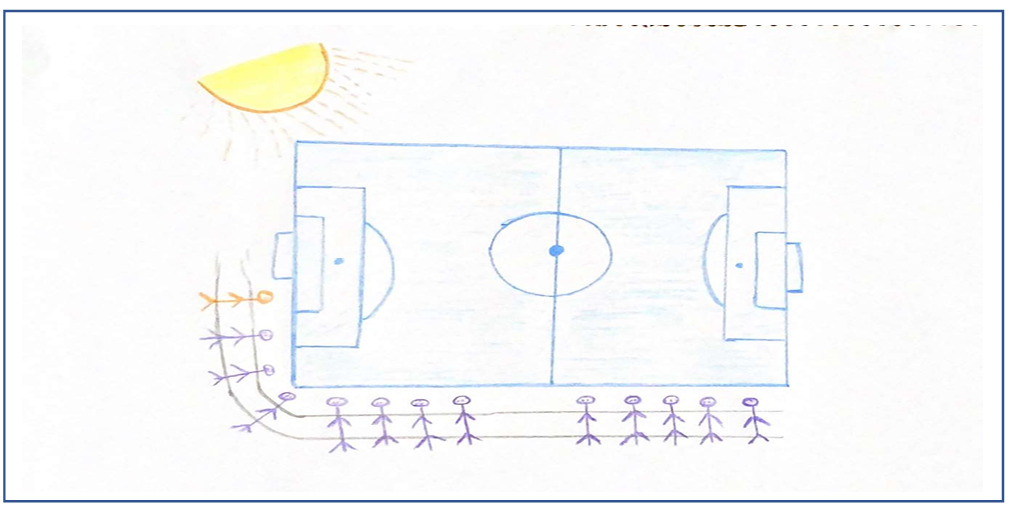


**Fig 3:** Student Group Drawing from Public School Workshop at Table C. This drawing shows a playground at the center, surrounded by lines indicating that the male students prefer engaging in the activity two to three times, and in the presence of their teacher, who acts as a leader. Additionally, the sun drawn at the top of the page symbolizes hot weather as a barrier.


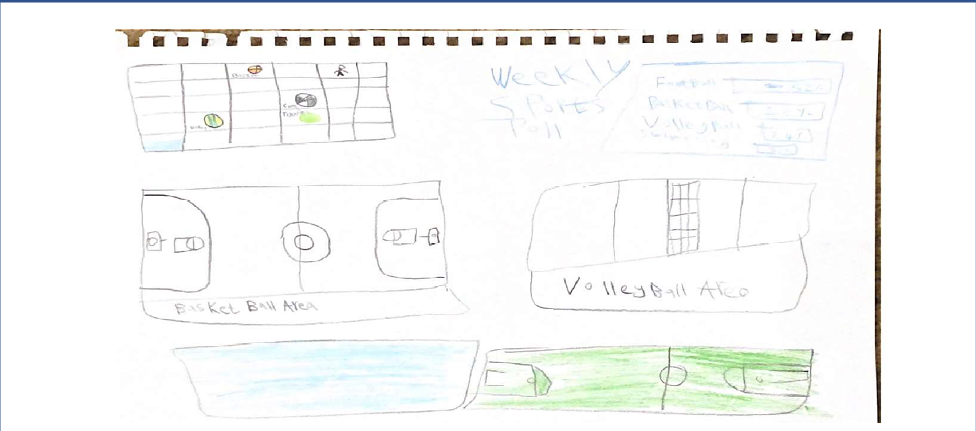


**.**

**Fig 3:** Student Group Drawing from Private School Workshop at Table B. This drawing shows the schedule, and they placed three sessions a week. Also, they presented four different activities they wanted to do in each session: football, swimming, basketball, and volleyball.


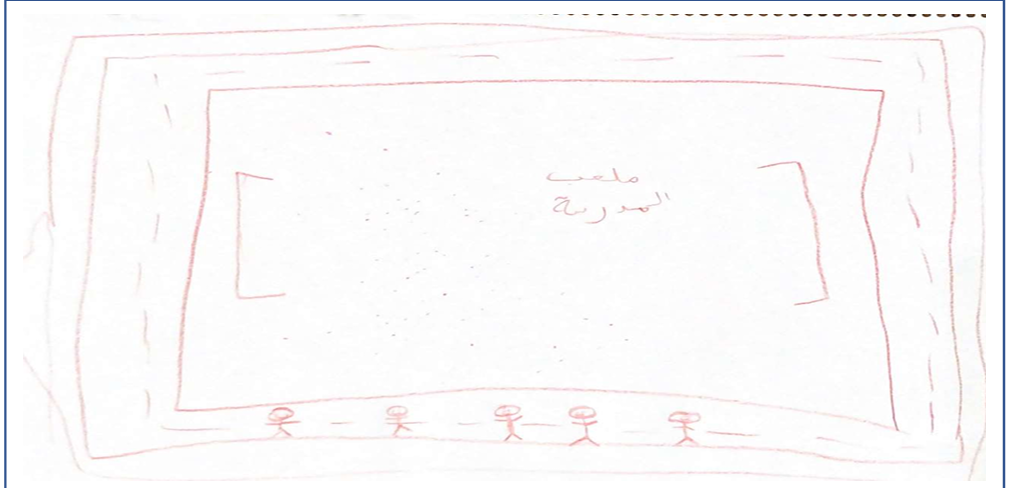


**Fig 4:** Student Group Drawing from Public School Workshop at Table B. This drawing presented by four students and it features a playground in the centre, labelled in Arabic as "school playground." Around it, a line indicates that the students will enjoy walking around the playground.


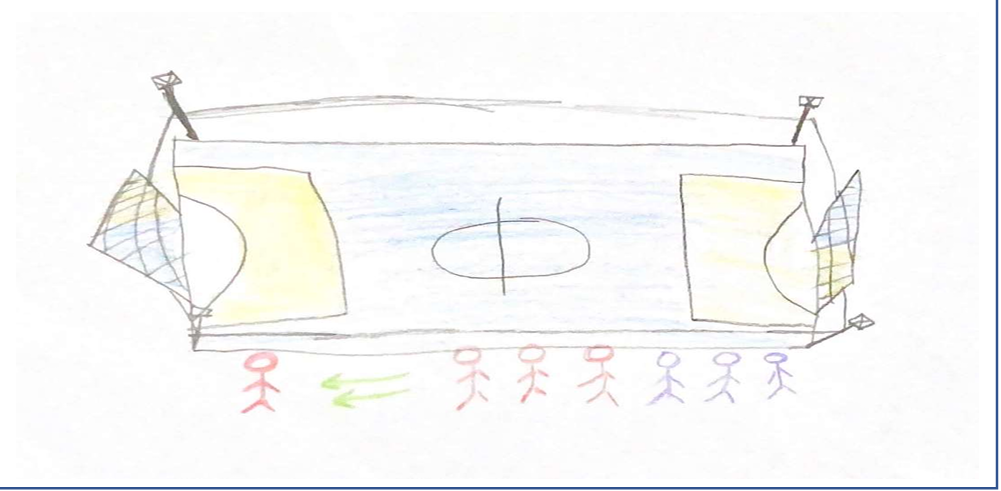


**Fig 5:** Student Group Drawing from Public School Workshop at Table D. This drawing presented by four male students and it depicts a playground in the centre. The students are shown walking around the playground, in front of their leader (teacher) and they are behind.

.


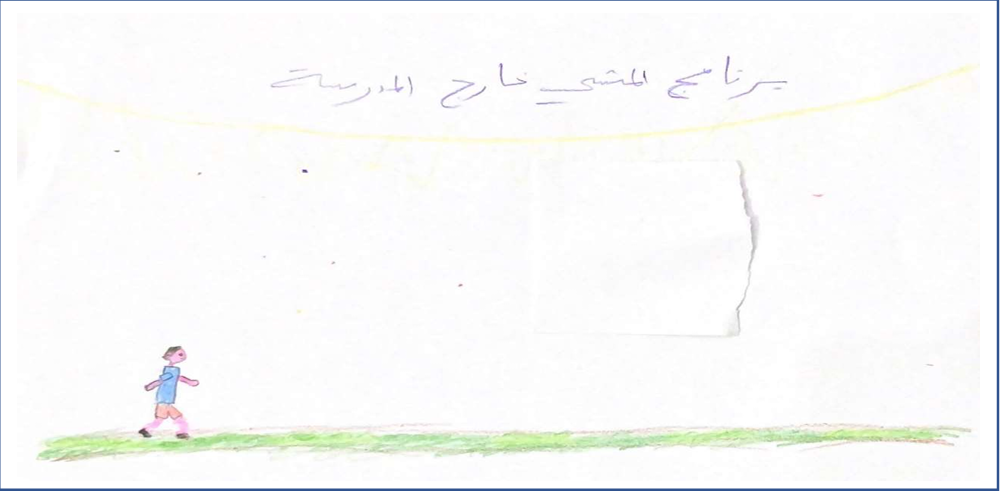


**Fig 6:** Student Group Drawing from Private School Workshop at Table E. This drawing was presented by three male students and it shows a green path and a boy walking and is written in Arabic at the top (walking programme outside the school).


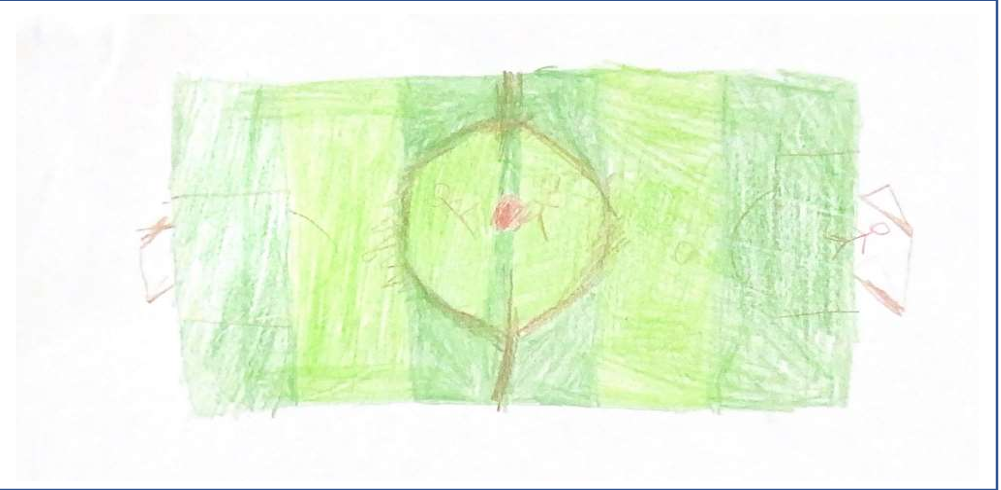


**Fig 7:** Student Group Drawing from Private School Workshop at Table C. This drawing was presented by four male students and it shows the playground and students playing football.


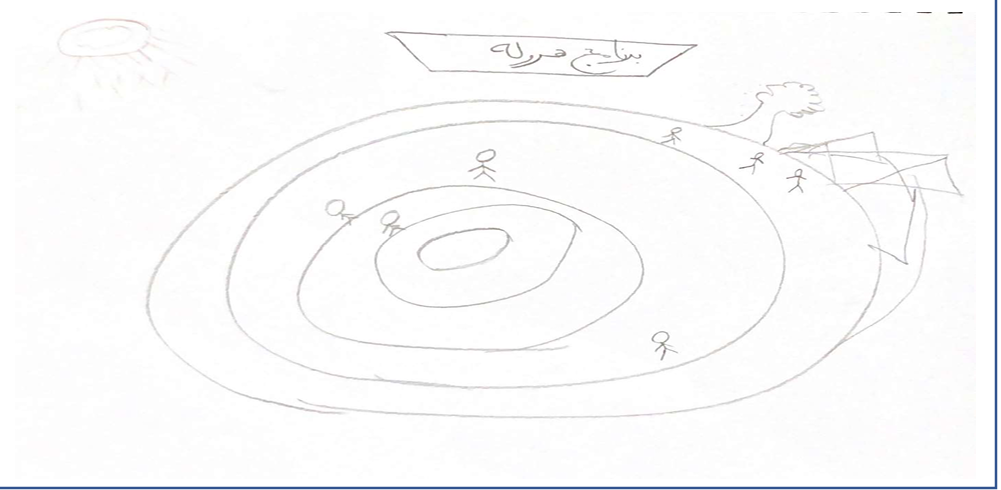


**Fig 8:** Student Group Drawing from Private School Workshop at Table A. This drawing presented by four male students and it depicts students jogging in circles in open areas, with a depiction of the sun on the upper left, symbolising outdoor jogging within the school premises. At the top, "jogging programme" is written in Arabic. The students expressed a desire to participate in this activity two to three times a week.

.


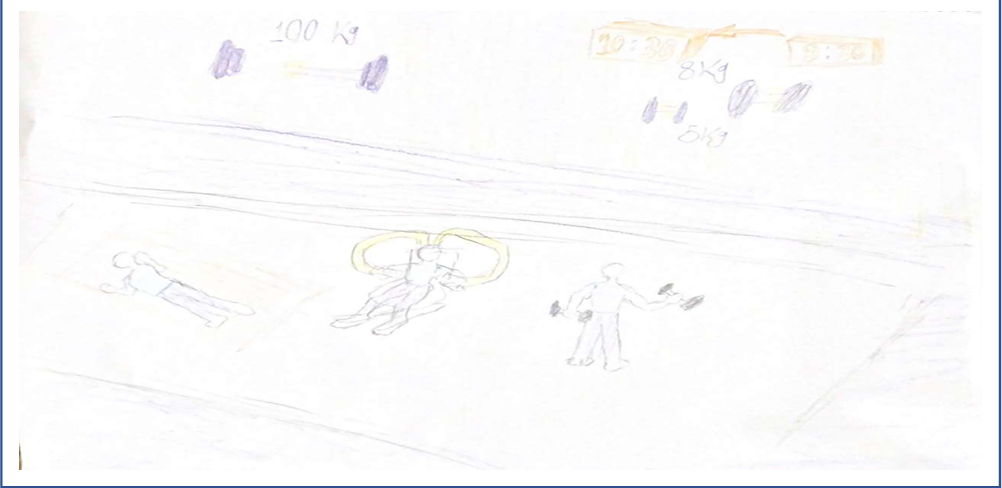


**Fig 9:** Student Group Drawing from Private School Workshop at Table F. This drawing presented by three male students and it depicts three students training in the gym, lifting weights. At the top, gym equipment is shown, with one weight labelled 100 kg and another 8 kg. Additionally, the time for the session is noted at the top as from 9:30 to 10:30.
