# Supplementary material for: Exploring barriers and facilitators to physical activity among children in Saudi Arabian schools: A qualitative study
Source: PLoS One. 2025 Sep 15;20(9):e0329600. doi: 10.1371/journal.pone.0329600 (PMC12435728; doi:10.1371/journal.pone.0329600)
Supplement: S5 File — (DOCX) [file pone.0329600.s006.docx]

Inclusivity in global research

PLOS’ policy on inclusivity in global research aims to improve transparency in the reporting of research performed outside of researchers’ own country or community and ensures that PLOS publications reporting global research adhere to high standards for research ethics and authorship. Authors of relevant research articles may be asked to complete the questionnaire below, which outlines ethical, cultural, and scientific considerations specific to inclusivity in global research. This questionnaire may be requested when researchers have travelled to a different country to conduct research, if research uses samples collected in another country, research with Indigenous populations or their lands, or if research is on cultural artefacts. Researchers travelling to another country solely to use laboratory equipment will not normally be required to complete the questionnaire. However, the questionnaire can be requested at the journal’s discretion for any submission – if you have been requested to complete this questionnaire by the PLOS journal you submitted to, please do so.

Please complete the questionnaire below and include this as a Supporting Information file with your manuscript. Note that if your paper is accepted for publication, this checklist will be published with your article in the supporting information files. Please ensure that you reference the checklist in the main body of your manuscript. We suggest adding a subsection ‘Inclusivity in global research’ to your Methods section and adding the following sentence: “Additional information regarding the ethical, cultural, and scientific considerations specific to inclusivity in global research is included in the Supporting Information (SX Checklist)”

The questions have been designed to be applicable to a wide range of study types, and there are subsections for both human subjects research and non-human subjects research. If any of the questions are not relevant to your research please mark them as “N/A” as appropriate.

**Ethical considerations, permits and authorship**

*This section is applicable to all research types.*

Provide details as to who granted permissions and/or consent for the study to take place in the Methods section of your manuscript. This should include the names of **all** ethics boards, governmental organizations, community leaders or other bodies that provided approval for the study. If individuals provided approval refer to these people by their role or title but do not list their name(s).

Reported on page number: 7 and 11.

If there were any deviations from the study protocol after approval was obtained please provide details of these changes in the Methods section of your manuscript.
Did this study involve local collaborators that are residents of the country where the research was conducted or members of the community studied? If you do not have any authors from said communities, please provide an explanation for this below.

Reported on page number: There were no deviations from the study protocol after ethical approval was obtained

No local collaborators were included as co-authors. However, the lead author is a Saudi national and a member of the community in which the research was conducted. As such, he possesses deep cultural and contextual knowledge, which informed all aspects of the research, including study design, data collection, and interpretation. The study also involved extensive engagement with local participants and stakeholders to ensure cultural sensitivity and relevance.

Everyone listed as an author should meet PLOS’ criteria for authorship and all individuals who meet these criteria should be included in the author byline, rather than the acknowledgements. For further information please see the journal’s Authorship Policy.

**Human subjects research (e.g. health research, medical research, cross-cultural psychology)**

Did you obtain written informed consent from a representative of the local community or region before the research took place? How did you establish who speaks for the community? Details of written informed consent obtained from study participants should be reported separately in the Methods section of your manuscript.

While no formal written informed consent was obtained from a designated representative of the community, official approval to conduct the research was obtained from the Ministry of Education office in Jeddah, Saudi Arabia. This served as institutional permission to engage with schools and participants in the region. Additionally, ethical approval was granted by the University of Sheffield’s ethics committee, and written informed consent was obtained from all individual participants prior to data collection, as detailed in the Methods section.

How did members of the local community provide input on the aims of the research investigation, its methodology, and its anticipated outcome(s)?

Members of the local community provided indirect input through their roles as participants in the study. The research aims and methodology were informed by the lead author’s personal and professional knowledge of the local context, as well as existing literature on physical activity in Saudi schools. During data collection, participants were encouraged to share their perspectives on barriers, facilitators, and potential strategies to enhance physical activity in schools. These insights helped shape the interpretation of findings and anticipated outcomes.

When engaging with the local community, how did you ensure that the informed consent documents and other materials could be understood by local stakeholders?

All informed consent documents and study materials were translated from English into Arabic, the native language of the study population. A back-translation process was conducted to ensure accuracy and conceptual equivalence. The lead author, a native Arabic speaker and member of the local community, ensured cultural appropriateness and clarity of all materials. In addition, the interview questions were piloted with individuals similar to the target participants to assess comprehension, flow, and cultural sensitivity. This process ensured that local stakeholders could fully understand the materials before providing informed consent.

Will the findings of the research be made available in an understandable format to stakeholders in the community where the study was conducted (e.g. via a presentation, summary report, copies of publications, etc.)? Please provide details of how this will be achieved.

Yes. A comprehensive dissemination strategy has been developed to ensure that the research findings are

accessible and useful to stakeholders in the community.

For schools and participants, workshops will be held with principals, teachers, and students to present the findings and discuss practical recommendations. Educational materials, including Arabic-language pamphlets, guides, and toolkits, will be developed and distributed to participating schools. In addition, summary reports will be shared with students, parents, and school staff to ensure transparency and show appreciation for their involvement.

Community meetings will be hosted at participating schools, and findings will also be shared via school websites and social media platforms to reach a broader audience. A policy brief will be submitted to the Ministry of Education, and a presentation is planned at the Ministry’s headquarters in Riyadh, where the lead author has already initiated contact with a senior advisor.

These steps will ensure that findings are not only published in academic outlets but also made understandable and actionable for those who contributed to and are affected by the research

**Non-human subjects research using specimens/ animals collected as part of the study, or those housed in archival collections. Examples include archaeology, paleontology, botany and zoology.**

Did the permission you obtained from a local authority to perform the study include an agreement on access to outputs and benefit sharing? This may include procedures to enable fair distribution of the benefits and resources arising from the research performed. Please include any details of Prior Informed Consent and Benefit Sharing Agreements obtained. These may be required by field-specific regulations, for example the Convention on Biological Diversity (CBD) and the associated Nagoya Protocol.

N/A

If the material used in your study was imported, please A) provide the year it was imported and B) indicate whether permits were obtained to import/export the materials used, C) provide details of any permits obtained. If this information is not available, please indicate this.

N/A

If you used archival specimens, please state how the material used in your study was acquired by the institute it is held in and provide details of any permits obtained for the original excavations/ sample collection. If this information is not available, please indicate this.

N/A

How was the potential cultural significance of the materials collected in your study to local communities considered in your research design? Were Indigenous peoples and/or local researchers and institutions involved with archaeological excavations / collection of specimens? If so, please provide a description of their involvement.

N/A

If your manuscript includes photographs of human remains please indicate whether authors obtained permission from descendants or affiliated cultural communities to do so.

N/A
